# Olaparib synergy screen reveals Exemestane induces replication stress in triple‐negative breast cancer

**DOI:** 10.1002/1878-0261.70093

**Published:** 2025-07-13

**Authors:** Nur Aininie Yusoh, Liping Su, Suet Lin Chia, Xiaohe Tian, Haslina Ahmad, Martin R. Gill

**Affiliations:** ^1^ Department of Radiology, Huaxi MR Research Center (HMRRC), Institution of Radiology and Medical Imaging West China Hospital of Sichuan University, Sichuan University Chengdu China; ^2^ UPM‐MAKNA Cancer Research Laboratory, Institute of Bioscience Universiti Putra Malaysia Serdang Malaysia; ^3^ Department of Microbiology, Faculty of Biotechnology and Biomolecular Science Universiti Putra Malaysia Serdang Malaysia; ^4^ Malaysia Genome and Vaccine Institute National Institutes of Biotechnology Malaysia Kajang Malaysia; ^5^ Department of Chemistry, Faculty of Science Universiti Putra Malaysia Serdang Malaysia; ^6^ Department of Chemistry, Faculty of Science and Engineering Swansea University UK

**Keywords:** aromatase inhibitor, drug combination, PARP inhibitor, TNBC

## Abstract

Triple‐negative breast cancer (TNBC) remains the breast cancer subtype with the poorest prognosis. While PARP inhibitors (PARPi) effectively target BRCA1/2‐mutant TNBCs via synthetic lethality, most TNBCs are BRCA1/2 wild‐type. Synergistic drug combinations may expand PARPi efficacy to BRCA‐proficient TNBC. To identify new PARPi combinations, we screened a library of 166 FDA‐approved oncology drugs for synergy with Olaparib in TNBC cells. We found that Exemestane, an aromatase inhibitor, synergized with Olaparib, significantly decreasing IC_50_ values and clonogenicity while increasing DNA damage and apoptosis. The mechanistic basis for this synergy was rationalized by the previously unreported ability of Exemestane to induce replication stress via reactive oxygen species (ROS) generation and oxidative stress. This combination had low cytotoxicity toward normal breast epithelial cells, and Exemestane has no reported severe toxicity as a monotherapy. The combination of Olaparib and Exemestane was able to achieve enhanced tumor growth inhibition in a murine xenograft model, greater than either drug employed as a single agent, and GO and KEGG enrichment analysis indicated alterations in pathways associated with cell death in response to Exemestane and Olaparib treatment.

## Graphical abstract

Screening 166 FDA‐approved anticancer drugs identifies the aromatase inhibitor Exemestane as a synergistic partner of PARP inhibitor Olaparib in BRCA‐proficient triple‐negative breast cancer. Exemestane induces ROS‐mediated replication stress, enhancing DNA damage and apoptosis alongside Olaparib. This previously unreported effect suggests Exemestane could extend the use of PARP inhibitors to BRCA‐proficient TNBC, addressing a critical gap in current treatment options.

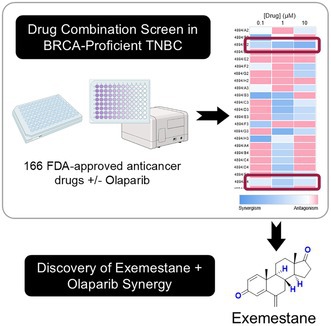

**Abbreviations**
8‐oxo‐dG: 8‐oxo‐2′‐deoxyguanosine
BET: bromodomain and extra‐terminal motif
BP: biological processes
BRCA1/2: breast cancer susceptibility genes 1/2
CC: cellular components
CCNE1: cyclin E1
CDK4: cyclin‐dependent kinase 4
CI: combination index
DCF: 2′,7′‐dichlorofluorescein
DCFH‐DA: 2′,7′‐dichlorodihydrofluorescein diacetate
DDR: DNA damage response
DEGs: differentially expressed genes
DSBs: DNA double‐strand breaks
EdU: 5‐ethynyl‐2′‐deoxyuridine
ER: estrogen receptor
EXE: Exemestane
GO: Gene Ontology
hEGF: human EGF
HER2: human epidermal growth factor receptor type 2
HR: homologous recombination
KEGG: Kyoto Encyclopedia of Genes and Genomes
MF: molecular functions
MTT: thiazolyl blue tetrazolium bromide
NAC: *N*‐acetylcysteine
NER: nucleotide excision repair
OLAP: Olaparib
PDGFRB: platelet‐derived growth factor receptor beta
PI3K: phosphatidylinositol‐3 kinase
PR: progesterone receptor
PTEN: phosphatase and tensin homolog
RPTOR: regulatory associated protein of MTOR complex 1
siNC: negative control siRNA
SSBs: single‐strand breaks
ssDNA: single‐stranded DNA
TNBC: triple‐negative breast cancer
WRN: Werner syndrome protein

## 1 Introduction

Comprising 15–20% of all breast cancer cases [1], triple‐negative breast cancer (TNBC) is an aggressive subtype characterized by the lack of expression of estrogen receptor (ER), progesterone receptor (PR), and human epidermal growth factor receptor type 2 (HER2). While non‐TNBC patients have benefited from major improvements in personalized therapy such as estrogen therapy or HER2‐targeted treatment, only a small number of novel therapeutic agents have gained approval for TNBC [2]. These limited treatment options combined with the heterogeneous nature and high metastatic potential of TNBC often lead to early relapse and a poorer prognosis compared to other subtypes [3].

A recent advancement in targeted therapeutics is poly(ADP‐ribose) polymerase (PARP) inhibitors (PARPi), which have shown promise as anticancer therapeutics, particularly toward BRCA‐mutated BC [4, 5, 6]. Mechanistically, this relies on the principle that when PARP enzymatic activities are inhibited, DNA single‐strand breaks (SSBs) are converted to DNA double‐strand breaks (DSBs). If the homologous recombination (HR) repair pathway is deficient, such as in those patients with BRCA mutations, DSBs remain unrepaired, leading to genome instability and/or cell death. However, in BRCA‐proficient cells, this DSB damage is repaired via the HR pathway and thus, PARP inhibition alone may not necessarily induce cell death. Hence, the efficacy of PARPi monotherapy is limited to BRCA‐mutated BC patients and the majority of TNBC, particularly those without BRCA1/2 mutations (80–90% of TNBC patients) [7, 8], still do not benefit from PARPi.

Drug combinations employing synergistic pairs can achieve improved antitumor responses with lower concentrations of single agents and overcome resistance [9, 10]. For PARPis, rational combination selection focusing on complementary mechanisms has attracted considerable interest as a potential method by which to expand the scope of PARPis, including towards BRCA‐proficient cancers [11]. This has seen numerous DNA‐damaging agents achieve synergy with PARPi, including platinum drugs [12, 13], doxorubicin [14], and gemcitabine [15], while targeting alternative DNA repair pathways has also achieved success in this capacity [16, 17]. An alternative method to identify drug combinations is unbiased (or hypothesis‐free) screening [18]. By utilizing chemical libraries alongside the drug of interest, this has the advantage of allowing for serendipitous discoveries and can facilitate greater understanding of the polypharmacology of drugs, where subsequent target deconvolution studies of a newly identified combination can reveal novel, sometimes unexpected, mechanisms of action [19]. Moreover, when an FDA‐approved library is employed, clinical translation may be accelerated. Despite this, such unbiased synergy screens involving PARPi are relatively rare. The validity of this approach may be demonstrated by Lui et al. [20], who reported the high‐throughput drug combination screening of PARPi rucaparib alongside a compound library consisting of 395 FDA‐approved and investigational compounds. Strikingly, the authors found that the effect of Bromodomain and extra‐terminal motif (BET) inhibitors was enhanced by rucaparib in patient‐derived ovarian cancer cells and, encouragingly, that this effect occurred irrespective of homologous recombination deficiencies. In addition to this, a recent high‐throughput screening study successfully identified BRCA1‐suppressing agents that sensitize BRCA1 wild‐type cancer cells to PARPi [21].

In this study, we performed an unbiased drug synergy screen employing Olaparib, the most successful PARPi to date, alongside an FDA‐approved oncology drug library to identify synergistic combinations. We uncovered that Exemestane and Olaparib are synergistic in BRCA‐proficient TNBC cells, likely due to the hitherto unknown ability of Exemestane to generate replication stress, characterize the mechanism of action of drug synergy, and validate this combination in a murine model. Altogether, our study provides a novel combination of Olaparib active toward TNBC *in vitro* and *in vivo* and reveals a new mechanistic function of Exemestane.

## 2 Materials and methods

### 2.1 Materials and reagents

Antibodies for p‐Chk1 (Ser345), p‐ATR (Thr1989), total ATR, p‐ATM (Ser 1981), total ATM, p‐histone H2AX (Ser139), RPA1, β‐actin, and HRP‐linked secondary antibody were purchased from Cell Signaling Technology (CST; Danvers, MA, USA). Antibodies for total Chk1 and Alexa‐Fluor 488‐conjugated secondary antibodies were purchased from Abcam (Cambridge, UK). Antibodies for p‐AKT (Ser473), total AKT, total ATM, p‐Chk2 (Thr68), total Chk2, PARP1, and GAPDH were purchased from Proteintech (Rosemont, IL, USA). Antibodies for p‐ATM (Ser1981) were also purchased from Affinity Biosciences (Cincinnati, Ohio, USA). Click‐iT® 5‐ethynyl‐2′‐deoxyuridine (EdU) imaging kit was purchased from Invitrogen (ThermoFisher Scientific, Waltham, MA, USA). 2′,7′‐dichlorodihydrofluorescein diacetate (DCFH‐DA) and Rosup were purchased from Solarbio (Beijing, China). 8‐oxo‐dG antibody, Olaparib, Exemestane, *N*‐acetylcysteine (NAC), Berzosertib, and Ipatasertib were purchased from MedChemExpress (MCE; Monmouth Junction, NJ, USA). Cisplatin was purchased from Sigma‐Aldrich (St. Louis, MI, USA). All other materials or reagents were purchased from Sigma‐Aldrich and ThermoFisher Scientific, unless otherwise specified. Stock solutions of Olaparib, Exemestane, Berzosertib, and Ipatasertib were prepared at 10 mm in 100% dimethyl sulfoxide (DMSO) and were kept at −20 °C. Stock solutions of cisplatin (2 mm) and NAC (10 mm) were prepared in phosphate‐buffered saline (PBS). 166 FDA‐approved anticancer drugs were obtained from the Drug Synthesis and Chemistry Branch, Developmental Therapeutics Program, Division of Cancer Treatment and Diagnosis, National Cancer Institute (NCI) DTP Repository (Rockville, MD, USA) as a plated set of compounds for research use. The details of each drug obtained from NCI DTP Repository, including their CAS number and molecular weight (MW), are described in Table S1. The approved anticancer drug set was provided in 96‐well plates formatted with each well containing an individual compound at a volume of 20 μL and a concentration of 10 mm dissolved in 100% DMSO. These plated sets were kept at −20 °C. Compounds lacking long‐term solubility in DMSO were suspended just before dispensing to avoid precipitation. Additionally, sub‐stock solutions of the library were prepared at 0.1 and 1 mm to support low‐concentration screening formats. All stock solutions were further diluted using PBS or Dulbecco's modified Eagle's medium (DMEM). The final DMSO concentration employed in the cell studies was < 0.1%.

### 2.2 Cell lines and culture conditions

The human breast cancer cell lines MDA‐MB‐231 (RRID: CVCL_0062), HCC1937 (RRID: CVCL_0290), MCF7 (RRID: CVCL_0031) were cultured in DMEM supplemented with 10% fetal bovine serum (FBS) and 1% penicillin/streptomycin (P/S) antibiotic. The human breast cancer cell line MDA‐MB‐436 (RRID: CVCL_0623) was cultured in DMEM supplemented with 15% FBS, 1% P/S antibiotic, l‐glutamine, sodium pyruvate, 10 μg·mL^−1^ insulin, and 16 μg·mL^−1^ glutathione. The MCF10A (RRID: CVCL_0598) normal breast cell line was cultured in DMEM supplemented with 5% horse serum, 0.5 μg·mL^−1^ hydrocortisone, 20 ng·mL^−1^ recombinant human EGF (hEGF), 10 μg·mL^−1^ insulin, and 1% P/S antibiotic. Cells were cultured as monolayers and maintained in a cell culture incubator at 37 °C under a humidified atmosphere containing 5% carbon dioxide (CO_2_). They were routinely subcultured with trypsin approximately twice a week at 80–90% confluency. Unless stated otherwise, all treatments were started 24 h after seeding. All cell lines used in this study were authenticated within the past 3 years using short tandem repeat (STR) profiling with the PowerPlex 18D System (Promega, Madison, WI, USA), following the manufacturers' protocols. Cell line identities were verified against the Cellosaurus database. All experiments were conducted using mycoplasma‐free cells, confirmed by regular mycoplasma testing.

### 2.3 MTT assay

MDA‐MB‐231 TNBC, MDA‐MB‐436 TNBC, HCC1937 TNBC, MCF7, and MCF10A normal cells were seeded in 96‐well plates (1 × 10^4^ cells/well for MDA‐MB‐231, MDA‐MB‐436, and MCF10A cells, and 5 × 10^3^ cells/well for MCF7 cells) and were treated as stated in the main text. For the drug combination screen in MDA‐MB‐231 cells, the working solution of each drug treatment at 10 μm and the subsequent 10‐fold dilutions were prepared. Single drugs as well as combinations were administered for 24 h. Each experimental 96‐well plate contained drug treatments, each in triplicate. The negative control (untreated cells, 0% inhibition) and positive control (Triton X‐100, 100% inhibition or complete cell death) were included in each plate. Following treatment, solutions were removed, thiazolyl blue tetrazolium bromide (MTT, 0.5 mg·mL^−1^) reagent was added to the cells, and plates were incubated for 3 h. The reduced purple formazan crystals were solubilized with 100 μL of DMSO, and the absorbance at 570 nm (620 nm as the reference wavelength) was measured using a microplate reader (Tecan Infinite F50, Tecan Group Ltd., Mannedorf, Switzerland). The half‐maximal inhibitory concentration (IC_50_) for inhibiting cell viability for each compound was derived from the dose–response curves using graphpad prism version 9.00 for Windows, GraphPad Software, La Jolla, CA, USA, www.graphpad.com.

### 2.4 Drug interaction analysis

For drug interaction analysis, dose–effect curves for mono‐ and co‐treatments were generated from MTT assay data, and the combination index (CI) values for each combination were calculated using calcusyn and compusyn software (Biosoft, Cambridge, UK) as established by Chou and Talalay [22, 23]. CI < 0.9 indicates synergism, CI = 0.9–1.0 indicates additive, and CI > 1 indicates antagonism. graphpad prism Software was used to generate a three‐color scale based on CI values obtained, where synergism is represented by blue, additive by white, and antagonism by red. The colors of each CI value were interpolated between these constraints accordingly. Ten hit compounds were isolated from the initial primary screen for further validation.

### 2.5 Clonogenic survival assay

MDA‐MB‐231, MDA‐MB‐436, HCC1937, and MCF7 cells were seeded (1 × 10^3^ cells/well) in 6‐well plates and were treated as stated in the main text. After treatment, solutions were removed, and cells were cultured in a compound‐free medium for 7–14 days to allow colony formation. Cells were then washed twice with 1× PBS, fixed with ice‐cold 100% methanol for 15–20 min at 4 °C, and stained with a 0.5% crystal violet solution for 20 min. The staining solution was washed with water, and images were photographed with a digital camera. Individual colonies were counted using imagej software (National Institutes of Health, NIH; USA), and the survival fraction was determined (normalized to controls).

### 2.6 Cell cycle analysis

MDA‐MB‐231 cells were seeded (3 × 10^5^ cells/well) in 6‐well plates and were treated as stated in the main text. Following treatment, cells were trypsinized and washed with 1× PBS twice. This was followed by fixation in ice‐cold 70% ethanol for at least overnight at 4 °C. After fixation, fixed cells were centrifuged (201 RCF, 5 min), and the resulting cell pellets were washed with 1× PBS twice. Samples were resuspended in 500 μL of 1× PBS containing 100 μg·mL^−1^ RNase A solution for 15 min. Samples were then stained with propidium iodide (PI) (20 μg·mL^−1^, in the dark) at 37 °C. After incubation, samples were stored on ice until data acquisition by flow cytometry using a NovoCyte flow cytometer and novoexpress software (Agilent Technologies, CA, USA). For each sample, a minimum of 10 000 cells was counted.

### 2.7 Apoptosis annexin V‐FITC/PI assay

MDA‐MB‐231 cells were seeded (3 × 10^5^ cells/well) in 6‐well plates and were treated as stated in the main text. After treatment, cells were trypsinized and washed with 1× phosphate‐buffered saline (PBS) twice. This was followed by the addition of 500 μL 1× binding buffer and 5 μL Annexin V‐FITC (Elabscience, Houston, TX, USA). The cell‐containing mixture was incubated for 20 min at RT. Five microliter of PI (20 μg·mL^−1^) was added before flow cytometric analysis using a flow cytometer, and results were analyzed using novoexpress software. For each sample, a minimum of 10 000 cells was counted.

### 2.8 Immunoblotting

MDA‐MB‐231 cells were seeded (8 × 10^5^ cells/dish) in a 60 mm cell culture dish, allowed to adhere for 24 h, and were treated as stated in the main text. Following treatment, cells were harvested and lysed in 1× RIPA (radioimmunoprecipitation assay) buffer (CST) supplemented with protease inhibitors and phosphatase inhibitors. Aliquots of cell lysates (40 μg total protein) were separated by sodium dodecyl sulfate/polyacrylamide gel electrophoresis (SDS/PAGE) using 4–20% Mini‐PROTEAN TGX precast protein gels (Bio‐Rad, Hercules, CA, USA or Beyotime, Jiangsu, China) and were transferred onto a nitrocellulose membrane using wet western blot transfer. Following this, the membrane was blocked in blocking buffer (5% BSA in TBS‐T (0.1% Tween 20 in 1× TBS)) for 1 h at RT. Membranes were then washed once with TBS‐T (5 min) and probed with primary antibodies in 5% BSA in TBS‐T solutions, overnight, at 4 °C. The primary antibodies used were: p‐Chk1 (Ser345) (1 : 500), total Chk1 (1 : 1000), p‐ATR (Thr1989) (1 : 500), total ATR (1 : 1000), p‐ATM (Ser 1981) (1 : 500), total ATM (1 : 1000), p‐histone H2AX (Ser139) (1 : 1000), p‐AKT (Ser473) (1 : 1000), total AKT (1 : 1000), p‐Chk2 (Thr68) (1 : 1000), total Chk2 (1 : 1000), PARP1 (1 : 1000) and GAPDH (1 : 10 000) and β‐actin (1 : 1000). Membranes were then washed with TBS‐T (3 × 5 min) and probed with a suitable horseradish peroxidase (HRP)‐conjugated secondary antibody in 5% BSA in TBS‐T solutions (1 : 3000) for 1 h at RT. Membranes were then washed (TBS‐T, 3 × 5 min) and were incubated with chemiluminescence substrates of SignalFire ECL reagent (CST) or WesternBright ECL HRP substrate (Advansta, San Jose, California, USA) or Immobilon Western Chemiluminescent HRP Substrate Immobilon (Sigma‐Aldrich) for 1 min at RT. Protein expression was visualized using a Syngene G:Box gel documentation system or ChemiDoc™ MP Imaging System. imagej software was used for densitometry data acquisition. β‐actin or GAPDH was used as a loading control.

### 2.9 Immunofluorescence

Cells were seeded in a 60 mm cell culture dish on 24 × 24 mm coverslips at a seeding density of 8 × 10^5^ cells/dish and were treated as stated in the main text. The solution was removed, cells were washed with ice‐cold 1× PBS twice, and fixed with 4% PFA (15 min, RT). After fixation, PFA was removed and cells were washed with ice‐cold 1× PBS twice. Cells were then permeabilized with Triton X‐100 (0.5% in PBS, 15 min, on ice) and washed with ice‐cold 1× PBS thrice. Samples were blocked with 3% BSA in PBS‐T (PBS with 0.1% Tween 20) for 1 h before incubation with the primary antibody (anti‐RPA1 [1 : 50] and anti‐8‐oxo‐dG [1 : 100]) diluted in 3% BSA in PBS‐T overnight, in a humid chamber at 4 °C. Following incubation, samples were washed in PBS‐T (3 × 5 min) and incubated with Alexa Fluor 488‐conjugated secondary antibodies (1/200 dilution in 3% BSA in PBS‐T) for 1 h, protected from light at RT. After further washing with PBS‐T (3 × 5 min), samples were co‐stained with DAPI (5 μg·mL^−1^, 2 min). Coverslips were mounted onto glass slides with antifade mounting medium and cells were visualized by confocal microscopy in a dark room. Microscopy images were processed, and the number of positively stained cells was counted using imagej software. A minimum of 200 nuclei were counted for each independent experiment.

### 2.10 EdU DNA labeling assay

MDA‐MB‐231 cells were seeded in a 6‐well plate at a density of 1 × 10^5^ cells/well and allowed to adhere for 24 h. Cells were treated as described in the main text, followed by the addition of 10 μm EdU labeling solution to each well and incubation for 2 h at 37 °C. After incubation, cells were fixed with 1 mL of 4% paraformaldehyde (PFA) in PBS for 20 min at RT and washed twice with 1 mL of 3% bovine serum albumin (BSA) in PBS. Permeabilization was performed using 1 mL of 0.1% Triton^®^ X‐100 in PBS for 20 min at RT, followed by two washes with 1 mL of 3% BSA in PBS. The Click‐iT^®^ reaction cocktail (0.5 mL) was added to each well, briefly rocked to ensure even distribution, and the plate was incubated for 30 min at RT in the dark. The reaction cocktail was then removed, and wells were washed once with 1 mL of 3% BSA in PBS. Subsequently, 1 mL of 1× Hoechst 33342 solution was added to each well and incubated for 30 min at RT, protected from light. Following incubation, the Hoechst 33342 solution was removed, and cells were washed twice with 1 mL of PBS. Finally, cells were resuspended in 1 mL of PBS and visualized by fluorescence microscopy in a dark room. Microscopy images were processed, and EdU‐positive cells were quantified using imagej software, with a minimum of 200 nuclei counted per independent experiment.

### 2.11 Determination of ROS levels

MDA‐MB‐231 cells were seeded in a 6‐well plate at a density of 1 × 10^5^ cells/well and allowed to adhere for 24 h. The cells were treated as described in the main text, with or without 5 mm NAC. After incubation, the medium was removed, and the cells were washed with 1× PBS. The cells were then incubated with 10 μm DCFH‐DA dye in serum‐free culture medium for 60 min at 37 °C in the dark. Following incubation, the DCFH‐DA solution was removed, and the cells were washed three times with serum‐free culture medium. The resulting DCF fluorescence was imaged using a fluorescence microscope. Microscopy images were processed, and ROS levels were quantified using imagej software.

### 2.12 Flow cytometry measurement for γH2AX

MDA‐MB‐231 cells were seeded at 3 × 10^5^ cells/well in 6‐well plates and were treated as stated in the main text. Following treatment, cells were trypsinized and washed with 1× PBS twice. Cells were then fixed with 4% PFA for 15 min at RT. Following fixation, cells were washed with 1× PBS and resuspended in 100 μL of 1× PBS. Thereafter, cells were permeabilized by adding ice‐cold 100% methanol slowly to pre‐chilled cells, while gently vortexing to a final concentration of 90% methanol and left at −20 °C for 2 h. Cells were then washed in excess 1× PBS and incubated with diluted primary antibody (γH2AX, 1 : 400) in antibody dilution buffer (0.5% BSA in PBS) overnight at 4 °C. Following incubation, cells were washed with antibody dilution buffer and incubated with diluted fluorochrome‐conjugated secondary antibody for 1 h at RT, in the dark (Alexa‐Fluor 488, 1 : 2000). Next, samples were washed with antibody dilution buffer and resuspended in 500 μL 1× PBS containing RNase A solution (10 μg·mL^−1^, 15 min, RT). Samples were then stained with PI (20 μg·mL^−1^, 30 min, in the dark). Samples were acquired and analyzed with a NovoCyte flow cytometer and novoexpress software. For each sample, a minimum of 10 000 cells was counted.

### 2.13 Small interfering RNA (siRNA)‐mediated knockdown of PARP1

MDA‐MB‐231 cells were seeded at ~ 50% confluency and incubated for 24 h prior to transfection. PARP1 knockdown was achieved using two previously validated small interfering RNA (siRNA) sequences targeting PARP1 (siPARP1#1 and siPARP1#2; Tsingke, Beijing, China). Cells were transfected in serum‐free medium using TSnanofect V2 transfection reagent (Tsingke) according to the manufacturer's instructions. A non‐targeting siRNA served as a negative control. Cells were harvested 48 h post‐transfection for downstream analyses. The efficiency of PARP1 knockdown was confirmed by western blotting. All subsequent functional experiments were conducted 48 h post‐transfection.

### 2.14 *In vivo* xenograft model

5 × 10^6^ MDA‐MB‐231 cells were injected subcutaneously into the right hind limb of female nude mice to establish a heterotopic tumor model (*n* = 5). Upon tumor formation after 20 days and when the tumor volume reaches about 70 mm^3^, the mice were randomly divided into four following groups (Control, Olaparib, Exemestane, and their combination, *n* = 5 per treatment group). Olaparib was administered at 50 mg·kg^−1^, and Exemestane was administered at 20 mg·kg^−1^, every 2 days for 30 days by intraperitoneal injection. Physiological saline was used as a solvent control. Tumor growth was assessed every 2 days for the following 30 days using vernier calipers, and tumor volume was calculated using the formula: tumor volume = (length × width^2^)/2. The body weight of the mice was recorded every 2 days for a total of 30 days. At the end of the 30‐day period, the mice were euthanized, 1 day after the last treatment, and the heart, liver, spleen, lungs, kidneys, and tumors were collected for further analysis. In this study, animal experiments were conducted in accordance with the protocol approved by the Animal Ethics Committee of West China Hospital of Sichuan University (approval no. 20230726001).

### 2.15 Histological and immunohistochemical (IHC) staining analysis

Tissue samples were fixed in 4% paraformaldehyde and embedded in paraffin. Paraffin blocks were sectioned into 5–6 μm thickness, placed onto slides, and stained with hematoxylin and eosin (H&E; ServiceBio, Wuhan, Hubei, China). For immunohistochemical staining analysis, slides were incubated with primary antibodies against Ki‐67 (GB111499, 1 : 1000; ServiceBio), CD31 (GB113151, 1 : 200; ServiceBio), and γH2AX (GB111841, 1 : 100; ServiceBio) overnight at 4 °C. Slides were then incubated with the anti‐rabbit HRP secondary antibody (GB23303, 1 : 200; ServiceBio) of the corresponding species of the primary antibody for 50 min and developed using 3,3′‐diaminobenzidine (DAB; ServiceBio). The slides of H&E and immunohistochemical staining assays were imaged using the SLIDEVIEW VS200 slide scanner (Olympus, Tokyo, Japan). Apoptosis was detected using a one‐step TUNEL apoptosis assay kit according to the manufacturer's instructions (Wuhan Servicebio Technology Co. Ltd., Hubei, China), and samples were observed under a Leica confocal microscopy (Stellaris 8). Nuclei were counterstained with hematoxylin or DAPI for IHC and the TUNEL reaction, respectively. Three images were randomly captured per slide, and the percentage of stained area was analyzed by imagej software.

### 2.16 Transcriptomics analysis

Briefly, tissue samples were subjected to LC–MS/MS analysis for omics evaluation. Transcriptome analyses were performed using the omics technology platform at Novogene Bioinformatics Technology Co., Ltd. (Beijing, China). Differentially expressed genes (DEGs) were analyzed using volcano plot analysis, clustered heat map analysis, Gene Ontology (GO) functional analysis, and Kyoto Encyclopedia of Genes and Genomes (KEGG) pathway enrichment analysis, all conducted with novomagic software and https://www.bioinformatics.com.cn, an online platform for data analysis and visualization.

### 2.17 Statistical analysis

Unless stated otherwise, the representation of figures and all data were statistically analyzed using graphpad prism software. All experiments were performed in triplicate and repeated three independent times (*n* = 3), unless specified otherwise. Each data point represents the mean value ± standard deviation (SD). Statistical significance between the groups was analyzed using one‐way analysis of variance (ANOVA). Differences were considered significant when the *P* values were less than 0.05.

## 3 Results

### 3.1 Exemestane shows synergism with Olaparib in TNBC cells

**Fig. 1:**
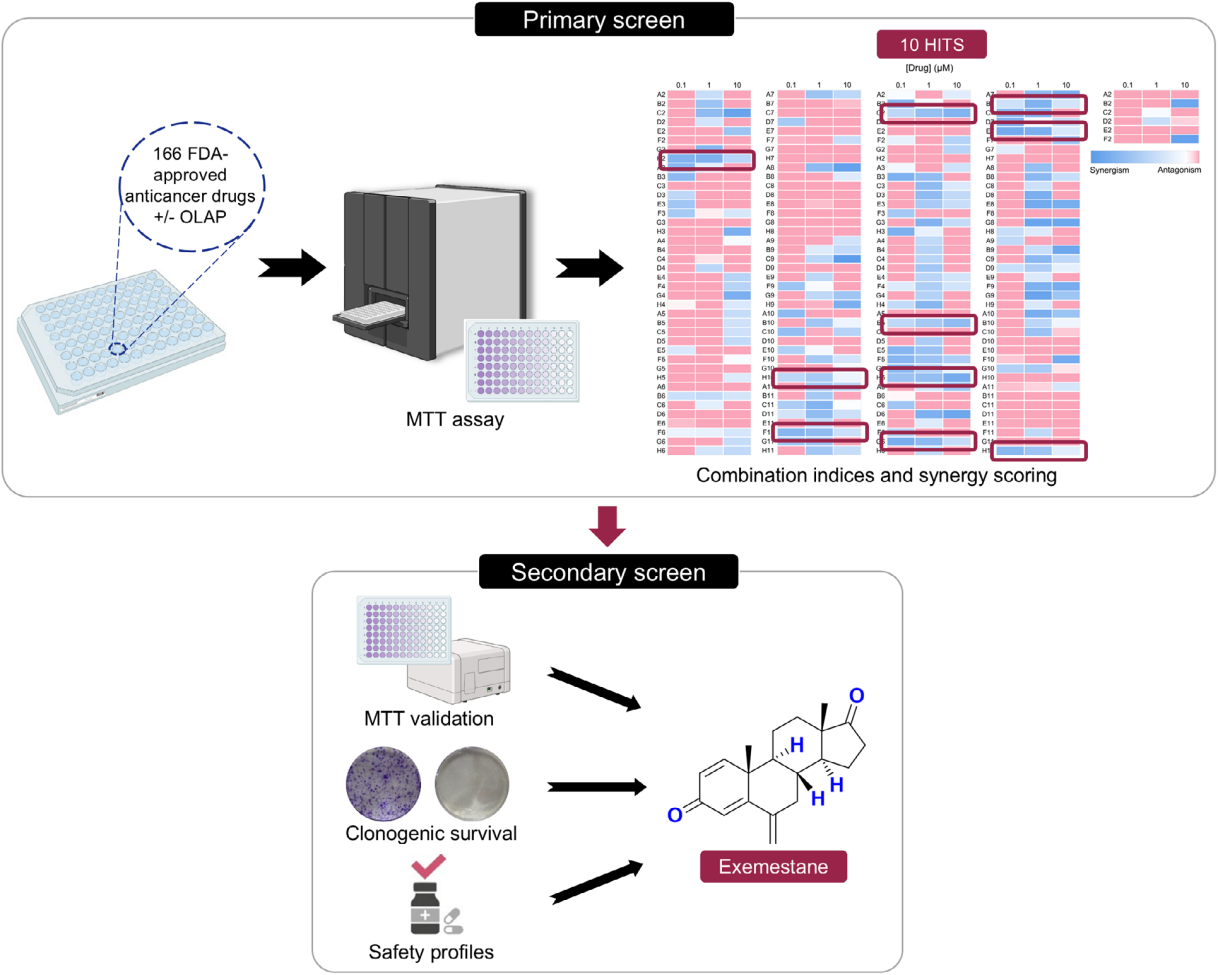
Schematic representation of the experimental steps implemented in the drug combination screening approach of Olaparib with a library of FDA‐approved oncology drugs in MDA‐MB‐231 TNBC cells. Icons used were created with BioRender.com. The heatmap of combination indices (CIs), cell viability data, clonogenic survival assay data, and literature search results from the drug combination screening study are shown in Tables S1–S4 and Figs S1–S3.

First, a drug combination screen was performed employing an FDA‐approved oncology drug library of 166 drugs (details within Table S1) and a non‐cytotoxic concentration of Olaparib in MDA‐MB‐231 TNBC cells (10 μm Olaparib; 24 h IC_50_ of Olaparib > 100 μm, Fig. S1), as depicted in Fig. 1. Resultant cell viabilities following treatment were determined by MTT assay and compared to single‐agent treatment conditions. Chou and Talalay combination index (CI) analysis was performed to determine synergy [22, 23]. This identified 10 compounds (16.9% of all drugs tested) that gave more than two synergistic CIs (CI < 0.9) when combined with Olaparib over the range of concentrations tested (Table S2), with substantial enhancement in cytotoxicity profiles compared to single agents alone (> 50% growth inhibition). These are chlorambucil, tamoxifen citrate, fludarabine phosphate, exemestane, zoledronic acid, abiraterone, omacetaxine mepesuccinate, panobinostat, plerixafor, and acalabrutinib. All hit compounds, except omacetaxine mepesuccinate and panobinostat, had no effect on cell viability when applied alone, with cell viabilities > 75% at 10 μm.

To verify hits, these 10 compounds were re‐tested at a greater concentration range (0.01–100 μm) in the absence and presence of a low concentration (10 μm) of Olaparib. Consistent with the initial findings, a substantial reduction in IC_50_ values (> 1.5‐ to > 200‐fold change in IC_50_ values of combination vs. single agent) was observed following co‐treatment with Olaparib compared to single agents alone, except for acalabrutinib, where IC_50_ concentrations of acalabrutinib alone or in combination are at > 100 μm (the maximum concentration tested; Table S3 and Fig. S2). In addition to the MTT assay, a long‐term clonogenic survival assay was conducted. All hit compounds as single agents possess a low impact on colony formation of MDA‐MB‐231 cells, where single agents Olaparib, exemestane, zoledronic acid, and acalabrutinib gave S.F. of 51.4%, 74.3%, 73.2%, and 81.7%, respectively (Fig. S3). Strikingly, exemestane, zoledronic acid, and acalabrutinib showed a significant (*P* < 0.05) reduction in colony formation when in combination with Olaparib (S.F. of 15.9%, 24.6% and 20.7% for exemestane, zoledronic acid and acalabrutinib, respectively).

**Fig. 2:**
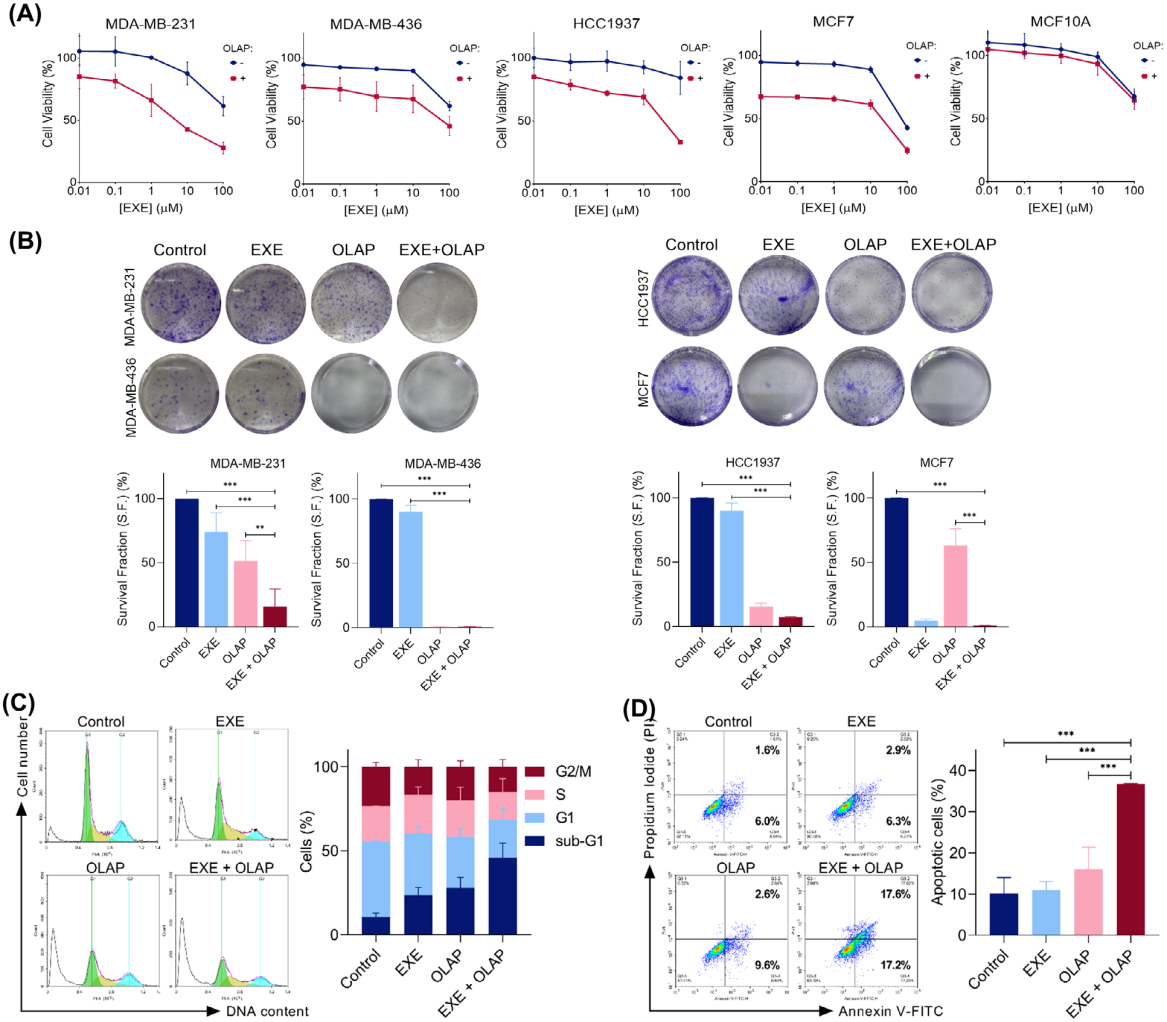
Exemestane synergized with Olaparib and led to increased apoptosis in MDA‐MB‐231 cells. (A) Cell viability of MDA‐MB‐231 TNBC, MDA‐MB‐436 TNBC, HCC1937 TNBC, MCF7 ER‐positive breast cancer, and MCF10A normal breast cells following 24 h treatment with concentration gradients of Exemestane, with and without 10 μm Olaparib, as determined by MTT assay. (B) Clonogenic survival assay of MDA‐MB‐231, MDA‐MB‐436, HCC1937, and MCF7 cells treated with Exemestane (EXE, 25 μm) and Olaparib (OLAP, 10 μm) alone or in combination (24 h treatment). Left, representative images; right, quantification of the percentage of survival factions following treatments. (C) Cell cycle distribution of MDA‐MB‐231 cells treated with Exemestane (25 μm) and Olaparib (10 μm) alone or in combination, 72 h treatment, as determined by PI staining and flow cytometry. Left, representative histograms; right, quantification of cell cycle phase. (D) Annexin V‐FITC assay of MDA‐MB‐231 cells treated as in (C). Left, representative scatterplots showing the percentage of cells in each quadrant; right, quantification of apoptotic cells (Q3–2 and Q3–4 quadrants) for each treatment condition. Data expressed as mean ± SD of at least three independent experiments. **P < 0.01 and ***P < 0.001 by ANOVA.

Finally, as the hit compounds are all FDA‐approved drugs, a literature search was carried out to identify their drug classes, the human safety profiles, and whether prior PARPi combination studies of these compounds have been reported. Surprisingly, most of the hit compounds are pharmacologically diverse from each other (Table S4). Moreover, studies have shown that chlorambucil, fludarabine phosphate, and panobinostat as monotherapies cause severe toxicity, limiting their use in clinics [24, 25, 26]. Interestingly, tamoxifen citrate (Clinicaltrials.gov identifier: NCT02093351) [27] and abiraterone (NCT03732820) [28] have been examined in combination with Olaparib in clinics. Therefore, these compounds were subsequently excluded. Collectively, Exemestane is identified as a new hit where no prior PARPi combinations have been reported with no reported severe toxicities as monotherapy. Moreover, significant decreases in cell survival verified the synergy observed between Olaparib and Exemestane, where a substantial reduction in IC_50_ values was observed (> 5.9‐fold change; IC_50_ values = > 100 μm vs. 16.8 ± 12.5 μm for combination vs. single agent; *P* < 0.05; Fig. 2A and Table S3). Besides, Olaparib and Exemestane combination treatment showed a significant decrease in clonogenicity (S.F. of 15.9% vs. 74.3% for combination vs. single agent; *P* < 0.01; Fig. 2B).

### 3.2 Exemestane plus Olaparib leads to elevated apoptosis in MDA‐MB‐231 cells

To evaluate the impact of the Exemestane and Olaparib combination across diverse breast cancer subtypes, we tested MDA‐MB‐436 and HCC1937 cells, both BRCA‐deficient TNBC models, alongside MCF7, an ER‐positive breast cancer cell line. The combination treatment demonstrated synergistic activity in all tested lines, significantly reducing cell viability compared to single agents at both 24 and 72 h (fold changes in 24 h IC_50_ values: > 1.07 for MDA‐MB‐436, > 1.09 for HCC1937, and 1.54 for MCF7; Fig. 2A, Fig. S4 and Table S5). Notably, Exemestane alone exhibited limited cytotoxicity in HCC1937 cells; however, its combination with Olaparib further decreased cell viability and colony formation, indicating additive or modest synergy even with wild‐type p53. Consistent with their HR deficiency, both MDA‐MB‐436 and HCC1937 cells showed marked loss of clonogenic survival upon Olaparib treatment (Fig. 2B). Conversely, ER‐positive MCF7 cells exhibited sensitivity to Exemestane monotherapy, reflecting their dependence on estrogen signaling. In comparison, the strongest synergy was observed in BRCA1‐proficient MDA‐MB‐231 TNBC cells, demonstrating the potential of this combination across varying genetic backgrounds. Moreover, to assess the cancer selectivity of the identified combination treatment, normal MCF10A breast epithelial cells were tested with Exemestane and Olaparib single agents or in combination. Normal MCF10A breast cells tolerated all combinations well, with cell viabilities > 70% for all concentrations tested (IC_50_s of > 100 μm for both single agent and combination; Fig. 2A). The minimal impact observed on normal breast epithelial cells indicates high cancer‐selective activity of this treatment. Next, the cell cycle distribution following treatment was examined using flow cytometric analysis, where cells were treated with Exemestane and Olaparib alone or in combination for 72 h, at drug doses optimized for synergy. Notably, co‐treatment with Exemestane and Olaparib caused a substantial increase in the proportion of sub‐G1 phase cells (35.1% increase compared to untreated controls; Fig. 2C), indicative of elevated apoptosis. Quantifying apoptosis by Annexin V‐FITC assay, single‐agent‐treated groups showed comparable levels of apoptotic cells in comparison to untreated control (< 5.8% increase; *P* > 0.05; Fig. 2D); however, significantly higher levels of Annexin V‐positive cells were apparent in the co‐treated group, indicative of high levels of apoptosis (26.6% increase in comparison to untreated control; *P* < 0.001).

### 3.3 Exemestane generates replication stress

**Fig. 3:**
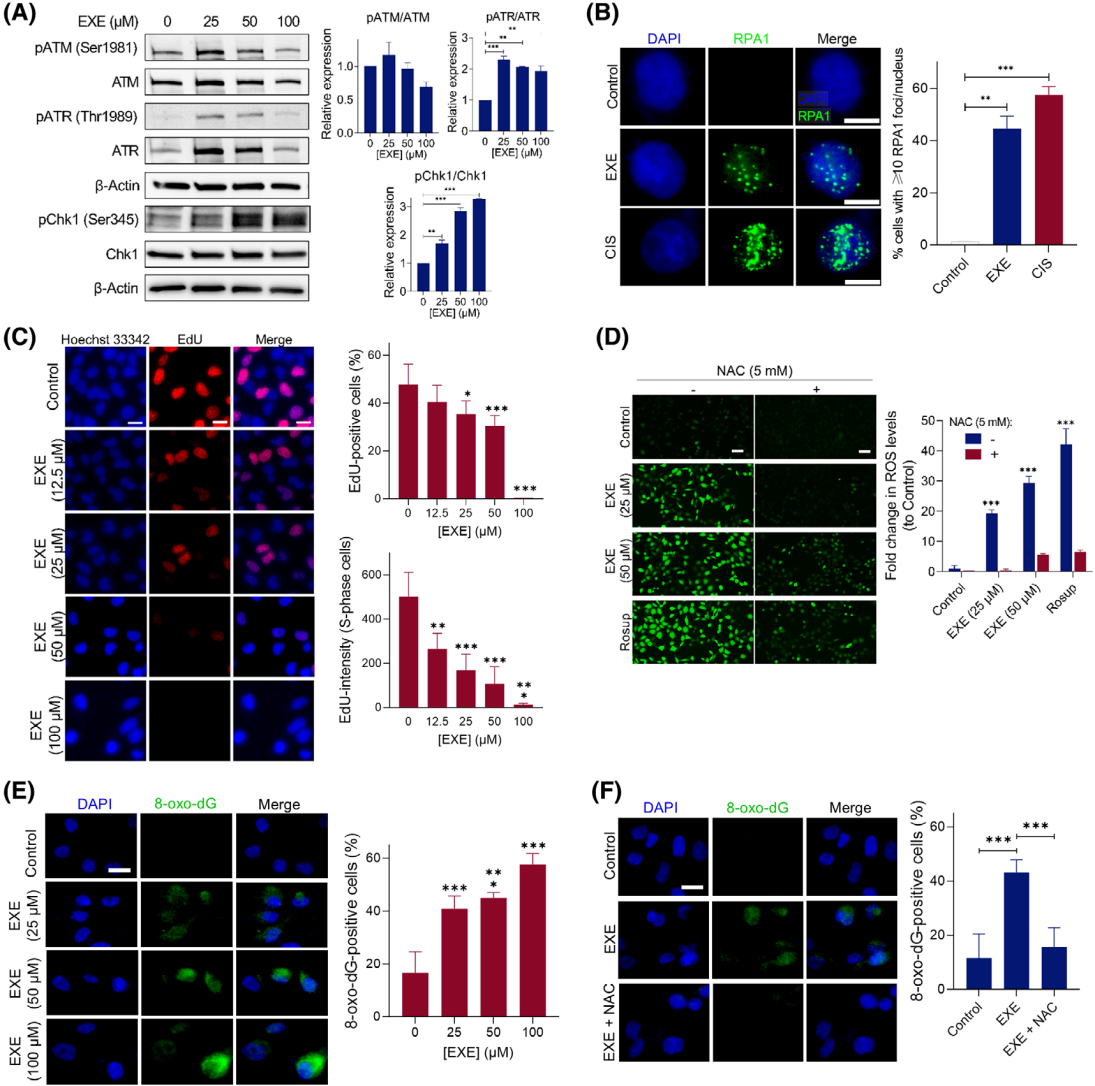
Exemestane as a single agent generates replication stress. (A) Western blot analysis of DDR activation following Exemestane treatment (25, 50 and 100 μm) for 3 h. Left, western blot images; right, quantification of protein bands from western blot images by densitometry. β‐Actin levels were monitored as a loading control. Uncropped blot images are shown in Fig. S6. Data expressed as mean ± SD of two independent experiments. **P < 0.01 and ***P < 0.001 by ANOVA. (B) RPA1 foci (green) formation in MDA‐MB‐231 cells nuclei following treatment with Exemestane (50 μm, 24 h). Cisplatin (CIS, 50 μm, 24 h) was employed as a positive control. Nuclear staining by DAPI (blue) also included. Scale bars = 10 μm. Left, representative images; right, quantification of cells with more than 10 RPA1 foci, where a minimum of 200 nuclei were counted for each independent experiment. Data expressed as mean ± SD of two independent experiments. *P < 0.05, **P < 0.01, and ***P < 0.001 by ANOVA. (C) 5‐Ethynyl‐2′‐deoxyuridine (EdU)‐positive cells (red) in MDA‐MB‐231 cells nuclei following treatment with a concentration gradient of Exemestane (24 h treatment). Nuclear staining by Hoechst 33342 (blue) also included. Scale bars = 20 μm. Left, representative images; right, quantification of the percentage of EdU‐positive cells (top) and EdU intensity in S‐phase cells (bottom) where a minimum of 200 nuclei were counted for each independent experiment. (D) Intracellular reactive oxygen species (ROS) levels measured using 2′,7′‐dichlorofluorescein (DCF) fluorescence in MDA‐MB‐231 cells following the indicated treatments for 24 h. ROS‐positive cells indicated by DCF (green). Rosup (500 μm, 1 h) was employed as a positive control. Scale bars = 50 μm. Data expressed as mean ± SD of two independent experiments. *P < 0.05, **P < 0.01, and ***P < 0.001 by ANOVA compared to the control group. (E, F) Representative images (left) and quantification (right) of 8‐oxo‐dG–positive nuclei (green) in MDA‐MB‐231 cells after 24 h of the indicated treatments. Nuclei were counterstained with DAPI (blue). Scale bars = 20 μm. Quantification represents the percentage of 8‐oxo‐dG–positive cells, with a minimum of 200 nuclei counted per independent experiment. Data are presented as mean ± SD from two independent experiments. Statistical significance was determined by one‐way ANOVA: *P < 0.001 vs. control.

Synergy with PARPi is often achieved by employing DNA‐damaging agents that generate SSBs or replication fork arrest [29, 30, 31]. Accordingly, to explore the basis for the observed synergy, the roles of Exemestane in DNA damage response (DDR) activation were investigated. MDA‐MB‐231 cells were treated with a concentration gradient of Exemestane for 3 h, and extracts were subjected to immunoblotting for several DDR signaling proteins, including activated (phosphorylated) p‐ATR (at Thr1989) and p‐ATM (at Ser1981), the two main upstream kinases in the signaling of DNA damage, and p‐Chk1 (Ser345). Following short‐term treatment with Exemestane, activation of the ATR signaling pathway was observed (1.9‐ to 2.3‐fold increase in p‐ATR/ATR for EXE vs. Control; *P* < 0.01; Fig. 3A). It was also revealed that p‐Chk1 increased significantly (1.7‐ to 3.3‐fold increase for EXE vs. Control; *P* < 0.01). In contrast, ATM phosphorylation (Ser1981) was not induced under these conditions. Flow cytometry revealed no major shifts in cell cycle distribution at 3 h (Fig. S5), consistent with early checkpoint activation rather than arrest. Although phosphorylation levels of ATM and ATR decreased following treatment with 100 μm Exemestane, correlating with reduced total protein levels, these results still indicate activation of ATR signaling. The reduction in total ATR and ATM may result from acute stress‐induced posttranslational modifications or enhanced proteasomal degradation, consistent with transient protein downregulation reported previously [32]. Interestingly, previous studies have shown that basal phosphorylated (p‐ATR) and endogenous levels of total ATR proteins increase in correlation with replication stress [33]. Strikingly, a significant increase in total RPA1 foci formation upon Exemestane treatment was observed, where RPA1 is a single‐stranded DNA (ssDNA) binding protein that stabilizes ssDNA during DNA damage repair and DNA replication (48.0% vs. 1.48% for EXE vs. Control; *P* < 0.01; Fig. 3B). These results indicate that Exemestane treatment leads to ATR/Chk1 signaling pathway activation, which responds to replication stress and increased DNA damage.

To investigate whether Exemestane inhibits DNA replication, a 5‐ethynyl‐2′‐deoxyuridine (EdU) labelling assay was performed to assess the proportion of cells actively synthesizing DNA during the S‐phase of the cell cycle. Noticeably, the Exemestane‐treated group showed a significant reduction in the percentage of EdU‐positive cells compared to the untreated control, indicating impaired S‐phase progression (30.5% vs. 47.8% for EXE [50 μm] vs. Control; *P* < 0.001; Fig. 3C). Total EdU intensity per S‐phase cell was also decreased, consistent with compromised DNA elongation. We next examined whether oxidative stress contributes to the observed replication stress. Exemestane treatment increased reactive oxygen species (ROS) levels (29.4‐fold increase in ROS levels for EXE (50 μm) vs. Control; *P* < 0.001), which was significantly reduced by N‐acetylcysteine (NAC) co‐treatment (Fig. 3D). To further substantiate the role of oxidative stress in the cytotoxic mechanism, we conducted 8‐oxo‐2′‐deoxyguanosine (8‐oxo‐dG) immunofluorescence staining to evaluate oxidative DNA damage. Exemestane‐treated cells showed a marked increase in nuclear 8‐oxo‐dG levels, which was significantly reversed by NAC co‐treatment (Fig. 3E,F), indicating a ROS‐dependent mechanism. These findings suggest that the synergistic cytotoxicity observed with the Exemestane and Olaparib combination may, at least in part, arise from oxidative DNA lesions induced by Exemestane and insufficiently repaired, thereby linking ROS generation to enhanced cell death. Together, these findings indicate that Exemestane induces replication‐associated DNA damage via ATR pathway activation and oxidative stress. Elevated 8‐oxo‐dG and RPA1 foci support S‐phase stress, rather than causing a definitive pre‐S‐phase arrest. At higher doses (≥ 100 μm), modest G2/M accumulation and sub‐G1 increases were observed (Fig. S5), consistent with downstream checkpoint activation and genotoxic stress.

### 3.4 Exemestane and Olaparib synergy accompanied by increased DNA DSB damage

**Fig. 4:**
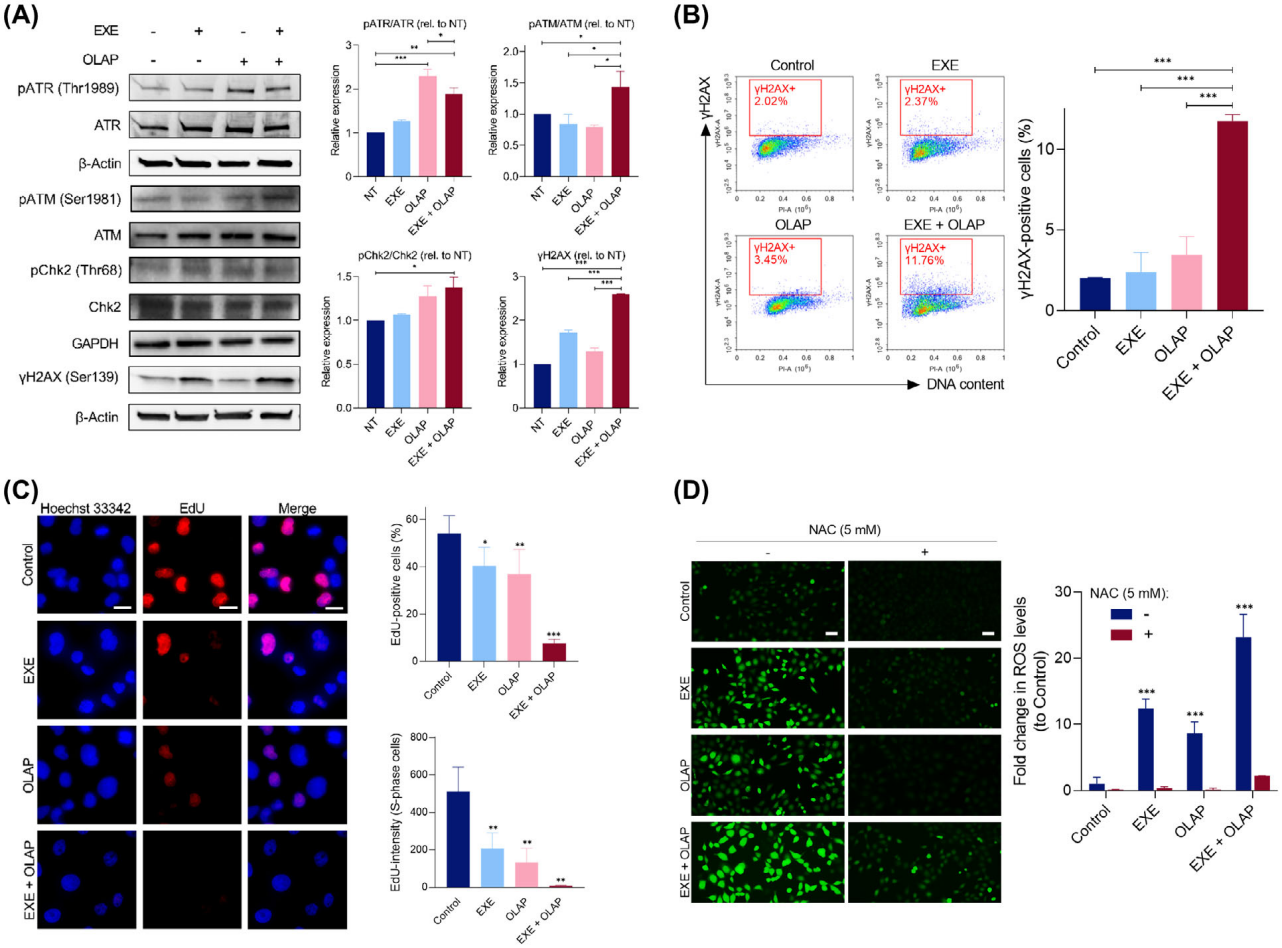
Exemestane and Olaparib synergy accompanied by increased DNA DSB damage. (A) Western blot analysis of DDR activation following treatment with Exemestane (25 μm) and Olaparib (10 μm) for 3 h. Left, western blot images; right, quantification of protein bands from western blot images by densitometry. β‐Actin levels were monitored as a loading control. NT, untreated control. Uncropped blot images are shown in Figs S7 and S8. Data expressed as mean ± SD of two independent experiments. *P < 0.05, **P < 0.01, and ***P < 0.001 by ANOVA. (B) γH2AX levels in cells treated as in (A) for 24 h, as determined by γH2AX/PI double staining. Left, the percentage of γH2AX‐positive cells (red gating) in each population where the *y*‐axis shows the level of γH2AX immunofluorescence and the *x*‐axis shows DNA content (PI). Data expressed as mean ± SD of three independent experiments. *P < 0.05, **P < 0.01, and ***P < 0.001 by ANOVA. (C) EdU‐positive cells (red) in MDA‐MB‐231 cells nuclei following treatment with a concentration gradient of Exemestane (24 h treatment). Nuclear staining by Hoechst 33342 (blue) also included. Scale bars = 20 μm. Left, representative images; right, quantification of the percentage of EdU‐positive cells (top) and EdU intensity in S‐phase cells (bottom) where a minimum of 200 nuclei were counted for each independent experiment. (D) Intracellular ROS levels measured using DCF fluorescence in MDA‐MB‐231 cells following the indicated treatments for 24 h. ROS‐positive cells indicated by DCF (green). Scale bars = 50 μm. Data expressed as mean ± SD of two independent experiments. *P < 0.05, **P < 0.01, and ***P < 0.001 by ANOVA compared to the control group.

We next evaluated the activation of the DDR signaling pathway after mono‐ and co‐treatment with Exemestane and Olaparib. As expected, Exemestane‐ and Olaparib‐treated groups showed a substantial increase in p‐ATR/ATR in MDA‐MB‐231 cells (Fig. 4A). Notably, a significant (*P* < 0.05) reduction in p‐ATR/ATR expression levels was observed upon co‐treatment with Exemestane and Olaparib compared to single agents or untreated control groups, suggesting that the combination of these drugs decreases the Olaparib‐ or Exemestane‐upregulation of p‐ATR/ATR. Notably, co‐treatment of Exemestane and Olaparib showed a significant increase in ATM/Chk2 signaling, along with elevated γH2AX (Ser139) levels, an early marker of DNA DSBs, indicating increased DNA damage upon combination treatment (1.7‐, 1.4‐, and 2.6‐fold increase for EXE + OLAP vs. NT for p‐ATM/ATM, p‐Chk2/Chk2 and γH2AX, respectively; *P* < 0.05). In addition to immunoblotting, the presence of DSBs was examined by monitoring γH2AX levels via flow cytometry after 24 h of drug treatment, a time in which apoptosis is not yet observed. Consistent with the initial finding, Exemestane and Olaparib as single agents showed comparable levels of γH2AX compared to untreated control, indicating low DSB damage generated by these compounds at sub‐cytotoxic concentrations (1.2‐fold for EXE vs. Control; 1.7‐fold for OLAP vs. NT; *P* > 0.05; Fig. 4B). Meanwhile, significantly greater γH2AX‐positive cells are apparent upon combination treatment compared to their single‐agent equivalents, indicating increased DNA DSB damage (5.8‐fold for EXE + OLAP vs. Control; *P* < 0.01). In addition, the combination treatment markedly reduced the proportion of EdU‐positive cells (7.7% vs. 54.2% in control; *P* < 0.001; Fig. 4C), indicating suppression of S‐phase entry. In addition, total EdU intensity per S‐phase cell was diminished, further supporting inhibition of DNA synthesis progression under combination treatment. This aligns with our earlier findings, which showed a substantial fraction of cells undergoing apoptosis. The combination also significantly increased ROS levels, which were mitigated by NAC, suggesting a link between oxidative DNA damage and the observed cell death (23.2‐fold increase in ROS levels for EXE + OLAP vs. Control; *P* < 0.001; Fig. 4D).

### 3.5 PARP1 knockdown confirms mechanistic basis of EXE/OLAP synergy

**Fig. 5:**
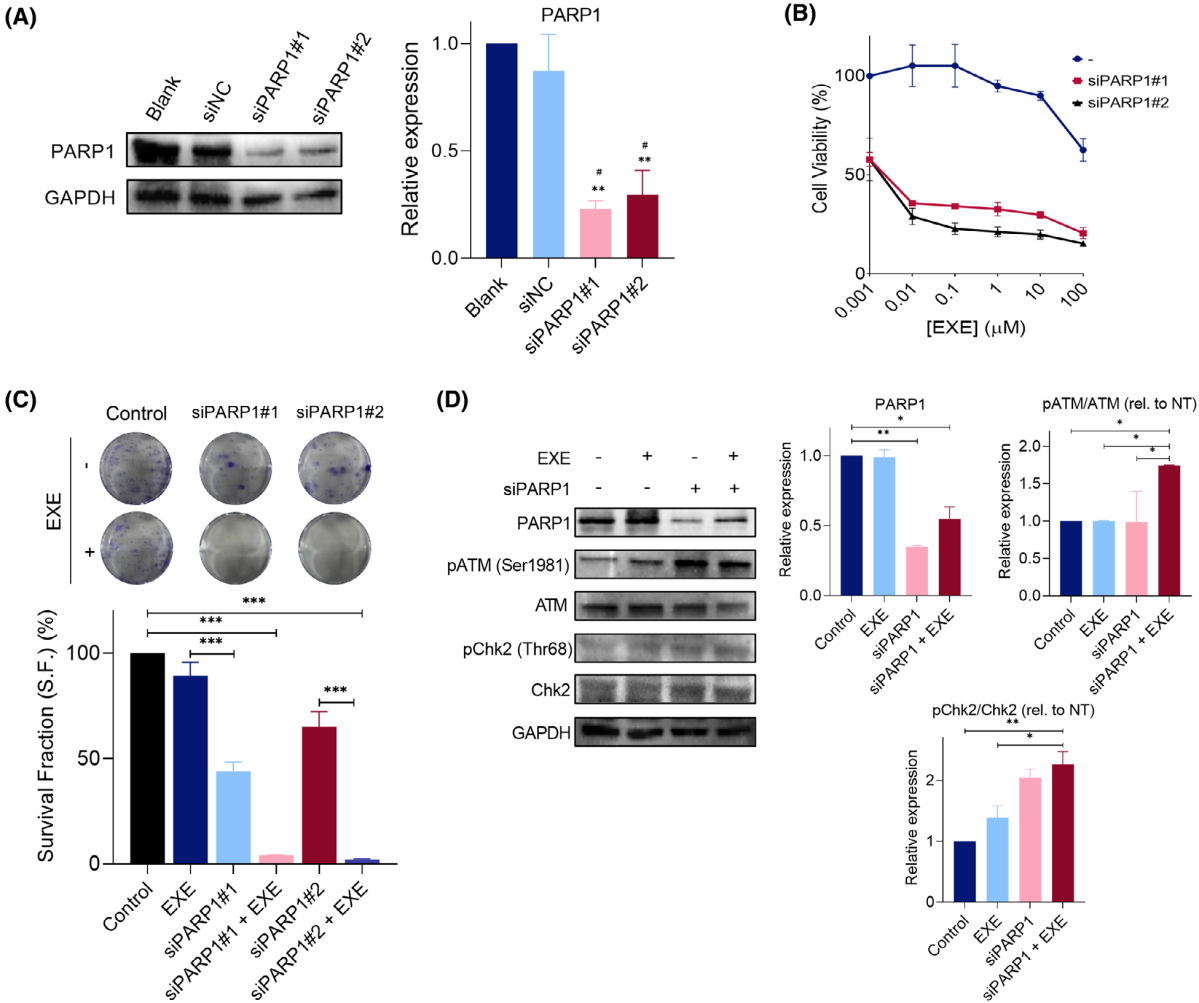
PARP1 knockdown confirms the mechanistic basis of EXE/OLAP synergy. (A) Western blot showing PARP1 levels following 48 h transfection with negative control siRNA (siNC) or siPARP1. Right: quantification by densitometry. GAPDH serves as loading control. NT, untreated. Uncropped blots in Fig. S9. Data are mean ± SD (*n* = 2); *P < 0.05, **P < 0.01 (*ANOVA comparison vs. Blank; ^#^ANOVA comparison vs. siNC). (B) Cell viability of MDA‐MB‐231 cells after 24 h Exemestane treatment with or without siPARP1, assessed by MTT assay. Data are mean ± SD (*n* = 3) (C) Clonogenic survival of MDA‐MB‐231 cells treated with Exemestane (25 μm) and/or siPARP1 for 24 h. Top: representative images; bottom: survival fraction quantification. (D) Western blot analysis of DDR markers following 3 h treatment with Exemestane (25 μm) with or without siPARP1. Right: densitometric quantification. GAPDH as loading control. NT, untreated. Uncropped blots in Fig. S10. Data are mean ± SD (*n* = 2); *P < 0.05, **P < 0.01, ***P < 0.001 (ANOVA).

To verify that the synergy observed between Exemestane and Olaparib is mediated through PARP inhibition rather than off‐target effects, we employed siRNA‐mediated knockdown of PARP1 in MDA‐MB‐231 cells. Western blot analysis confirmed effective PARP1 depletion following siRNA transfection (*P* < 0.05) (Fig. 5A). PARP1 silencing significantly enhanced Exemestane‐induced cytotoxicity, as demonstrated by reduced cell viability in MTT assays and impaired clonogenic survival (Fig. 5B,C). These findings support that the observed synergy with Olaparib is mediated through on‐target inhibition of PARP1. Mechanistically, Exemestane combined with PARP1 knockdown led to increased activation of DNA damage signaling, evidenced by elevated phosphorylation of ATM and Chk2 (1.7‐ to 2.3‐fold increase for siPARP1 + EXE vs. Control; *P* < 0.05), consistent with enhanced replication stress and DNA damage (Fig. 5D).

### 3.6 Exemestane enhances the effects of Olaparib *in vivo*

**Fig. 6:**
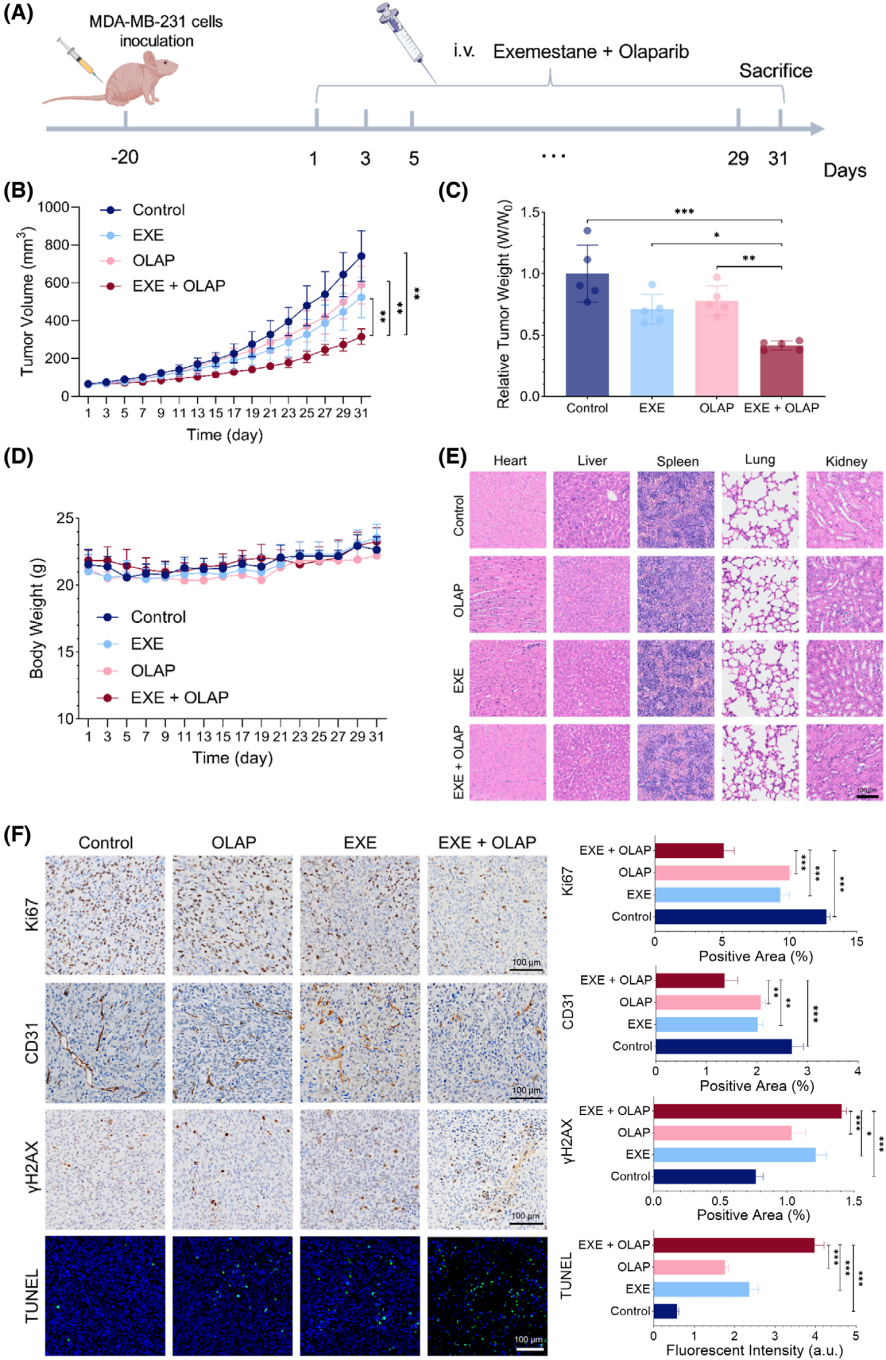
Exemestane enhances the effects of Olaparib *in vivo*. (A) Schematic diagram indicating MDA‐MB‐231 cells were injected into nude mice and the mice were subsequently treated with Olaparib (50 mg·kg^−1^ every 2 days for 30 days) and Exemestane (20 mg·kg^−1^ every 2 days for 30 days) alone or in combination at the indicated times. (B) Tumor volumes over 30 days of treatment with Olaparib and Exemestane alone or in combination (*n* = 5 per treatment group). (C) Relative tumor weight of different treatment groups as indicated (*n* = 5 per treatment group). (D) Body weight of mice throughout the study (*n* = 5 per treatment group). (E) Representative images of H&E‐staining of the tissue sections. Scale bars = 100 μm (*n* = 5 per treatment group). (F) Representative images of immunohistochemical staining for proliferation markers Ki67 (brown), vascular endothelial marker CD31 (brown) and DNA damage marker γH2AX (brown). TUNEL staining showing apoptotic cells (green) and nuclear staining (blue). Left, representative images of xenografts used for evaluation; right, quantification of the percentage of positive area. Scale bars = 100 μm. Data are shown as the mean ± SD (*n* = 5 per treatment group). *P < 0.05, **P < 0.01, and ***P < 0.001 by ANOVA.

To explore our findings *in vivo*, a MDA‐MB‐231 xenograft tumor model of nude mice was established to investigate the synergistic effect of Exemestane and Olaparib on the growth of MDA‐MB‐231 cells *in vivo* (Fig. 6A). After 30 days of treatment, Exemestane (20 mg·kg^−1^) and Olaparib (50 mg·kg^−1^) treatment alone resulted in no significant difference in tumor volume and tumor weight compared to the vehicle‐treated control mice (Fig. 6B,C). Strikingly, the combined treatment of Exemestane and Olaparib significantly (*P* < 0.05) inhibited tumor volume and tumor weight compared to single agents alone. Moreover, these findings were supported by the histologic analyses of the xenografts, which showed a significant (*P* < 0.05) decrease in proliferation markers Ki67 and vascular endothelial marker CD31, along with an increase in γH2AX, the DNA damage marker, for the combination group (Fig. 6F). Further TUNEL staining analysis revealed significantly (*P* < 0.001) greater areas of apoptotic cells (green) in the combination group compared to single agents alone and control. Encouragingly, no loss of body weight of mice was observed following administrations of Exemestane, Olaparib, and their combination at the tested dosages (Fig. 6D). H&E‐stained organs showed that these treatments did not cause significant damage to the heart, liver, spleen, lung, or kidney, suggesting the safety of this therapy (Fig. 6E).

### 3.7 Transcriptomic evaluations indicate alterations in pathways associated with cell death

**Fig. 7:**
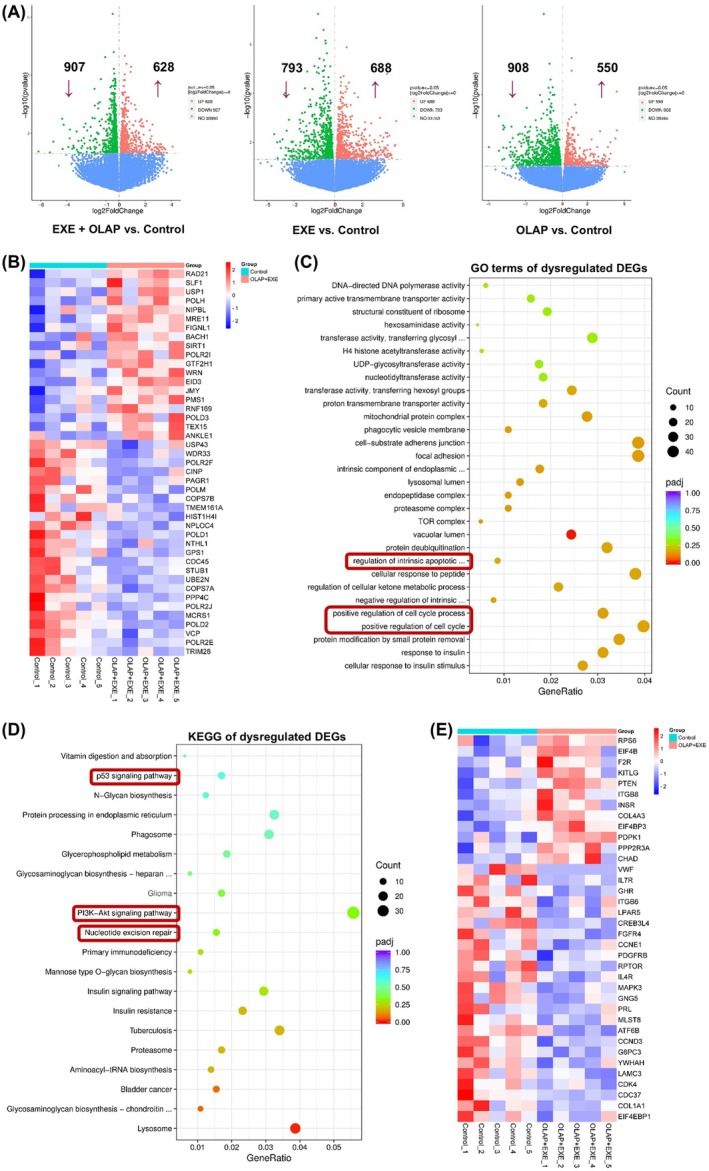
Transcriptomic evaluation following co‐treatment with Exemestane and Olaparib. (A) Volcano plot illustrating the differentially expressed genes (DEGs) across all treatment groups vs. control. Significantly upregulated (red) and downregulated (green) DEGs are shown. Log_2_ fold change (FC) = 0, *n* = 5 per group, and *P* < 0.05 is considered statistically significant. (B) Heatmap showing *z*‐score normalized intensities of significantly upregulated and downregulated DEGs involved in DNA repair pathways for EXE + OLAP vs. Control groups. *P* < 0.05 is considered statistically significant. (C) GO enrichment analysis of DEGs across three functional categories: biological processes (BP), cellular components (CC), and molecular functions (MF). (D) KEGG pathway enrichment analysis of DEGs in transcriptomes. The top 20 significantly enriched pathways are presented in order of enrichment score. Dot size represents gene count, and color represents the adjusted *P* value (*P*_adj_). (E) Heatmap showing *z*‐score normalized intensities of significantly upregulated and downregulated DEGs involved in PI3K‐Akt signaling pathways for EXE + OLAP vs. Control groups. *P* < 0.05 is considered statistically significant.

To further explore the mechanism by which PARP inhibition combined with Exemestane shows enhanced efficacy in MDA‐MB‐231 tumors, transcriptome sequencing was conducted to analyze the differentially expressed genes (DEGs) between the treatment groups. A volcano plot illustrated changes in transcriptome expression, revealing 628 significantly upregulated and 907 downregulated genes in the Exemestane and Olaparib co‐treated group compared to the control group (Fig. 7A). Since we previously showed that the co‐treated group exhibited increased DNA damage, we screened DEGs associated with DNA repair to analyze their expression. Interestingly, the genes RAD21, MRE11, SIRT1, and Werner syndrome protein (WRN) were significantly (*P* < 0.05) upregulated, potentially indicating heightened DNA damage repair capacity in this group (Fig. 7B) [34, 35]. Subsequently, GO functional analysis and KEGG pathway enrichment analysis were conducted to compare the co‐treated and control groups. GO analysis revealed that dysregulated DEGs were associated with pathways such as “regulation of cell cycle” and “regulation of intrinsic apoptosis signaling pathway in response to DNA damage” (Fig. 7C). Furthermore, KEGG analysis revealed enrichment of dysregulated DEGs in pathways including “phosphatidylinositol‐3 kinase (PI3K)‐Akt signaling pathways”, “nucleotide excision repair (NER)” and “P53 signaling pathways”, among others (Fig. 7D). Interestingly, the dysregulation of DEGs across all treatment groups reveals intriguing insights into the modulation of the PI3K‐Akt signaling pathway (Fig. S11), which plays a key role in cell survival and apoptosis [36, 37], and in drug resistance mechanisms [38, 39]. Olaparib treatment upregulates DEGs linked to the PI3K‐Akt pathway, suggesting the activation of cell survival mechanisms. Conversely, Exemestane treatment downregulates DEGs associated with the PI3K‐Akt pathway, potentially inhibiting this pro‐survival signaling cascade. Strikingly, the co‐treated group exhibited downregulation of DEGs in the PI3K‐Akt pathway, indicating a synergistic effect in suppressing this pathway. Supporting this observation, a clustering heatmap analysis of DEGs associated with the PI3K‐Akt signaling pathway revealed a significant (*P* < 0.05) increase in the expression of phosphatase and tensin homolog (PTEN) gene, a crucial tumor suppressor gene linked to negative regulation of PI3K‐Akt pathway (Fig. 7E). In addition, the genes cyclin E1 (CCNE1), platelet‐derived growth factor receptor beta (PDGFRB), regulatory associated protein of MTOR complex 1 (RPTOR), and cyclin‐dependent kinase 4 (CDK4) were found to be downregulated. These findings suggest that the combined inhibition by Exemestane and Olaparib disrupts key survival signals, leading to the suppression of the P13K–Akt pathway and thereby promoting cell death mechanisms via apoptosis in MDA‐MB‐231 tumors.

To investigate the potential involvement of the PI3K/AKT pathway indicated by our transcriptomic analysis, we assessed corresponding protein‐level changes upon treatment. Western blot analysis demonstrated a consistent increase in phosphorylated AKT (p‐AKT) following Olaparib treatment, whereas Exemestane alone or in combination with Olaparib caused a marked decrease in p‐AKT levels (Figs S12 and S13). These findings indicate concordance between transcriptional and posttranslational regulation, reinforcing that the combination treatment suppresses AKT signaling. To evaluate the functional relevance of this pathway, MDA‐MB‐231 cells were treated with the AKT inhibitor (AKTi) Ipatasertib. While AKT inhibition modestly reduced cell viability, the effect was not statistically significant. Clonogenic survival assays showed no further decrease in survival upon the addition of AKTi to the combination treatment compared to the combination alone, with no colonies observed in either group (Fig. S14). This lack of additive effect suggests that targeting AKT alone is insufficient to enhance cytotoxicity beyond that achieved by the drug combination. Additionally, we conducted MTT and clonogenic survival assays using the ATR inhibitor (ATRi) Berzosertib in combination with the Exemestane/Olaparib treatment. While the addition of ATRi modestly reduced cell viability compared to the combination alone or the AKTi/combination group (Fig. S14), this effect was not statistically significant. In clonogenic assays, no further reduction in colony formation was observed, suggesting that the combination treatment may already functionally suppress the ATR/Chk1 pathway. These findings indicate that additional ATR inhibition confers limited benefit, potentially due to pathway redundancy or compensatory mechanisms within the DNA damage response.

## 4 Discussion

The unbiased drug synergy screen employed in this study successfully isolated Exemestane, an aromatase inhibitor, as a synergistic pairing with Olaparib in BRCA‐proficient MDA‐MB‐231 TNBC cells. Our findings suggest that the combination therapy may have broad applicability across various breast cancer subtypes, including BRCA‐deficient TNBC and ER‐positive cells. Notably, the combination therapy demonstrated stronger synergy in BRCA wild‐type TNBC cells, underscoring its potential therapeutic advantage in this specific subtype. Exemestane blocks the synthesis of estrogen and is an effective and selective treatment for postmenopausal patients with hormone‐dependent breast cancers as monotherapy [40]. In patients with ER‐positive cancers, Exemestane has also been examined in combination with other therapeutics including antimetabolites [41], alkylating agents [42], kinase inhibitors [43, 44, 45], and immune checkpoint antibodies [46, 47]. Although Exemestane has not been examined in combination with PARPi, a phase I study has examined Olaparib with other endocrine therapy drugs, including anastrozole, letrozole, or tamoxifen, in advanced solid tumor patients and in ER‐positive breast cancer patients [27]. This combination was generally well tolerated, with no clinically relevant pharmacokinetic interactions identified.

Our results both identify a new Exemestane–Olaparib drug combination and indicate the basis for the observed synergy is that Exemestane induces replication stress in BRCA‐proficient cells—a previously unknown effect of the drug—and that the addition of a PARPi suppresses ATR activation, impairing DNA repair and resulting in the accumulation of cytotoxic DSBs, ultimately leading to apoptotic cell death. This serendipitous discovery into the polypharmacology of Exemestane validates our screening methodology. The Exemestane‐induced replication stress is a new discovery, and the precise basis for this is unknown. While it seems unlikely that Exemestane directly binds DNA, our findings suggest that this effect may result from ROS generation as a direct biological consequence of Exemestane treatment. This aligns with previous findings showing that, in addition to blocking estrogen biosynthesis, Exemestane was found to generate ROS, resulting in the growth arrest of breast cancer [48]. While it remains challenging to fully delineate whether Exemestane induces a late G1 arrest or replication stress during S‐phase, the combined evidence of ROS generation, oxidative DNA lesions, and replication‐associated damage suggests that cells progress into S‐phase under stress conditions. The observed G2/M accumulation at higher doses further supports a continuum of checkpoint responses rather than a discrete cell cycle block. These mechanistic features likely contribute to the enhanced sensitivity to PARP inhibition. To validate the on‐target basis of the observed synergy, PARP1 knockdown recapitulated the effects of Olaparib, enhancing Exemestane‐induced cytotoxicity and activating DNA damage signaling. These findings confirm that the synergy arises from PARP1 inhibition and support a model where Exemestane‐induced replication stress becomes lethal upon loss of PARP1‐mediated repair.

In this study, we focused on the ER‐negative, BRCA1‐proficient TNBC cell line MDA‐MB‐231 to investigate ER‐independent mechanisms underlying the synergy between exemestane and Olaparib. Given the absence of ER expression in this model, experiments were not conducted under hormone‐depleted conditions, as estrogen signaling is not anticipated to significantly influence exemestane response. Our findings therefore highlight a non‐canonical role for exemestane beyond estrogen suppression, potentially involving oxidative stress and DNA damage pathways. While we included preliminary data from the ER‐positive MCF7 cells to illustrate the absence of synergy in an estrogen‐dependent context, detailed mechanistic studies were not pursued in that model. We acknowledge the limitation of not using steroid‐stripped serum in the MCF7 setting [49, 50], and future work will be required to delineate the impact of estrogen availability on drug response in hormone‐responsive breast cancer models.

It is encouraging that the combination showed no cytotoxicity in normal breast epithelial cells and that this combination inhibited the tumor growth of BRCA‐proficient TNBC mouse xenograft model without noticeable signs of toxicity. The corresponding transcriptomic analysis provides further insights into the molecular mechanisms underlying the co‐treatment of Exemestane and Olaparib in MDA‐MB‐231 tumors. Subsequent GO functional analysis and enriched KEGG pathways analysis of DEGs revealed dysregulation in cell survival and cell death pathways, further supporting our findings. Specifically, the enriched KEGG pathways of PI3K‐Akt and P53 signaling influence apoptosis [37, 51], whereas nucleotide excision repair (NER) maintains genomic stability in response to DNA damage accumulation [52]. Interestingly, Olaparib treatment alone showed upregulation in the PI3K‐Akt pathway, which is crucial for cell survival, consistent with prior studies [53, 54]. Other studies have also demonstrated that Olaparib treatment promotes the expression of genes involved in DNA repair pathways [55]. In contrast, KEGG analysis suggests that Exemestane indirectly downregulates the PI3K‐Akt pathway in ER‐negative breast cancer (e.g., TNBC), although this observation requires additional experimental confirmation. Notably, downregulation of the PI3K‐Akt pathway was observed in the co‐treated group, indicating a synergistic effect in suppressing this pathway, possibly through p53 dysregulation [56]. These findings align with studies showing that inhibiting PI3K signaling (via BKM120) can sensitize BRCA‐proficient TNBC cells to Olaparib [57, 58]. In addition, a phase I trial has examined the combination of Olaparib with the AKT inhibitor, capivasertib, in patients with and without BRCA mutations [59]. While further investigation is needed to fully understand the interaction between Exemestane and Olaparib in BRCA‐proficient TNBC, these findings are consistent with our experimental results, which show extensive DNA damage beyond the repair capacity of the cells following co‐treatment. Consequently, the cells activate programmed cell death mechanisms to prevent the propagation of damaged DNA, leading to enhanced apoptosis. While this provides a plausible explanation for the increased DNA damage and enhanced apoptosis observed in experiments following treatment with Exemestane and Olaparib in MDA‐MB‐231 tumors, further investigation across a greater range of cancer types is required.

Moreover, optimal sequencing and combination strategies for Olaparib need to be further explored. Sequential therapy is often crucial in oncology, especially when combining different treatment modalities. Determining the most effective sequence can be challenging and requires thorough investigation through extensive preclinical and clinical trials. For instance, the PARTNER clinical trial assessing neoadjuvant Olaparib with carboplatin/paclitaxel treatment in BRCA‐proficient TNBC patients found no clinical benefit [60]. In another trial, carboplatin was administered prior to Olaparib in ovarian, breast, and uterine cancer patients with and without BRCA mutation [61]. Strikingly, clinical activity was also seen in subsets of BRCA‐proficient TNBC patients. These trials further underscore the necessity of refining the order in which treatments are given to maximize their effectiveness, as described in detail by Plana et al [62].

## 5 Conclusion

In summary, this drug screening study identified a novel synergistic combination of Exemestane and the PARPi Olaparib in BRCA‐wild‐type TNBC. These findings support the concept that PARPi‐based combination strategies may be a promising approach for aggressive TNBC, potentially extending the use of PARPi beyond BRCA‐deficient cancers. Notably, this newly identified synergy may expand the therapeutic potential of Exemestane to ER‐negative breast cancer, thereby benefiting a broader patient population. However, the optimal sequencing of Exemestane and Olaparib remains unknown, necessitating further investigation before clinical application.

## Conflict of interest

The authors declare no conflict of interest.

## Author contributions

MRG, HA, and XT conceived and designed the project. NAY conducted the *in vitro* experiments, and LS performed the *in vivo* experiments. NAY and LS handled data curation, data analysis, and data interpretation. NAY prepared the figures. Resources and supervision were provided by MRG, HA, XT, and SLC. NAY and MRG wrote the original draft of the manuscript. All authors revised and approved the final version of the manuscript.

## Peer review

The peer review history for this article is available at https://www.webofscience.com/api/gateway/wos/peer‐review/10.1002/1878‐0261.70093.

## Supporting information


**Fig. S1.** Cell viability of MCF10A, MDA‐MB‐231, MDA‐MB‐436, HCC1937, and MCF7 cells treated with Olaparib for 24 h (left) or 72 h (right), assessed by MTT assay.
**Fig. S2.** IC_50_ values of each compound alone or combined with Olaparib (10 μm) in MDA‐MB‐231 cells after 24 h treatment, based on MTT assay.
**Fig. S3.** Clonogenic survival assay of MDA‐MB‐231 cells treated with selected compounds (1 μm), Olaparib (10 μm), or their combination for 24 h.
**Fig. S4.** Cell viability of MDA‐MB‐231, MDA‐MB‐436, HCC1937, and MCF7 cells treated with Olaparib for 72 h, assessed by MTT assay.
**Fig. S5.** Cell cycle analysis of MDA‐MB‐231 cells following 24 h treatment with Exemestane (25–200 μm), as determined by PI staining and flow cytometry. Left: representative histograms; right: quantification of cell cycle phases.
**Fig. S6.** Uncropped western blot images corresponding to Fig. 3, with molecular weights (kDa) indicated.
**Fig. S7.** Uncropped western blot images corresponding to Fig. 4, with molecular weights (kDa) indicated.
**Fig. S8.** Additional uncropped western blot images corresponding to Fig. 4, with molecular weights (kDa) indicated.
**Fig. S9.** Uncropped western blot images corresponding to Fig. 5, with molecular weights (kDa) indicated.
**Fig. S10.** Additional uncropped western blot images corresponding to Fig. 5, with molecular weights (kDa) indicated.
**Fig. S11.** KEGG pathway enrichment analysis of differentially expressed genes in (A) Exemestane‐, (B) Olaparib‐, and (C) Exemestane + Olaparib‐treated groups compared to control.
**Fig. S12.** Western blot analysis of AKT signaling following 3 h treatment with Exemestane (25 μm) and Olaparib (10 μm).
**Fig. S13.** Uncropped western blot images corresponding to Fig. S12, with molecular weights (kDa) indicated.
**Fig. S14.** AKT and ATR inhibitors enhance Exemestane + Olaparib cytotoxicity in MDA‐MB‐231 cells.
**Table S1.** Details of the FDA‐approved drug set (166 compounds) obtained from the NCI.
**Table S2.** Combination indices (CIs) for the combinations of library compounds with Olaparib in MDA‐MB‐231 cells following 24 h treatment.
**Table S3.** IC_50_ values of each compound alone or in combination with Olaparib (10 μm) in MDA‐MB‐231 cells after 24 h treatment, as determined by MTT assay.
**Table S4.** Details of the 10 hit compounds, including drug classes, human safety profiles, and combination status with PARP inhibitors.
**Table S5.** IC_50_ values of Exemestane alone or in combination with Olaparib (10 μm) in MDA‐MB‐436 and MCF7 cells after 24 h treatment, as determined by MTT assay.

## Data Availability

The data that support the findings of this study are available in the figures and the Supporting Information of this article.
