# Supplementary material for: Olaparib synergy screen reveals Exemestane induces replication stress in triple‐negative breast cancer
Source: Mol Oncol. 2025 Jul 13;19(11):3387–408. doi: 10.1002/1878-0261.70093 (PMC12591312; doi:10.1002/1878-0261.70093)
Supplement: Supplementary file 1 — Fig. S1. Cell viability of MCF10A, MDA‐MB‐231, MDA‐MB‐436, HCC1937, and MCF7 cells treated with Olaparib for 24 h (left) or 72 h (right), assessed by MTT assay. Fig. S2. IC50 values of each compound alone or combined with Olaparib (10 μm) in MDA‐MB‐231 cells after 24 h treatment, based on MTT assay. Fig. S3. Clonogenic survival assay of MDA‐MB‐231 cells treated with selected compounds (1 μm), Olaparib (10 μm), or their combination for 24 h. Fig. S4. Cell viability of MDA‐MB‐231, MDA‐MB‐436, HCC1937, and MCF7 cells treated with Olaparib for 72 h, assessed by MTT assay. Fig. S5. Cell cycle analysis of MDA‐MB‐231 cells following 24 h treatment with Exemestane (25–200 μm), as determined by PI staining and flow cytometry. Left: representative histograms; right: quantification of cell cycle phases. Fig. S6. Uncropped western blot images corresponding to Fig. 3, with molecular weights (kDa) indicated. Fig. S7. Uncropped western blot images corresponding to Fig. 4, with molecular weights (kDa) indicated. Fig. S8. Additional uncropped western blot images corresponding to Fig. 4, with molecular weights (kDa) indicated. Fig. S9. Uncropped western blot images corresponding to Fig. 5, with molecular weights (kDa) indicated. Fig. S10. Additional uncropped western blot images corresponding to Fig. 5, with molecular weights (kDa) indicated. Fig. S11. KEGG pathway enrichment analysis of differentially expressed genes in (A) Exemestane‐, (B) Olaparib‐, and (C) Exemestane + Olaparib‐treated groups compared to control. Fig. S12. Western blot analysis of AKT signaling following 3 h treatment with Exemestane (25 μm) and Olaparib (10 μm). Fig. S13. Uncropped western blot images corresponding to Fig. S12, with molecular weights (kDa) indicated. Fig. S14. AKT and ATR inhibitors enhance Exemestane + Olaparib cytotoxicity in MDA‐MB‐231 cells. Table S1. Details of the FDA‐approved drug set (166 compounds) obtained from the NCI. Table S2. Combination indices (CIs) for the combinations of librar [file MOL2-19-3387-s001.docx]

**Olaparib synergy screen reveals Exemestane induces replication stress in triple-negative breast cancer**

Nur Aininie Yusoh^1,2,‡^, Liping Su^1,‡^, Suet Lin Chia^2,3,4^, Xiaohe Tian^1^*, Haslina Ahmad^2,5^* and Martin R. Gill^6^*

^1^Department of Radiology, Huaxi MR Research Center (HMRRC), Institution of Radiology and Medical Imaging, West China Hospital of Sichuan University, Sichuan University, Chengdu, Sichuan, China

^2^UPM-MAKNA Cancer Research Laboratory, Institute of Bioscience, Universiti Putra Malaysia, 43400 UPM Serdang, Selangor, Malaysia

^3^Department of Microbiology, Faculty of Biotechnology and Biomolecular Science, Universiti Putra Malaysia, 43400 UPM Serdang, Selangor, Malaysia

^4^Malaysia Genome and Vaccine Institute, National Institutes of Biotechnology Malaysia, Jalan Bangi, 43000 Kajang, Selangor, Malaysia

^5^Department of Chemistry, Faculty of Science, Universiti Putra Malaysia, 43400 UPM Serdang, Selangor, Malaysia

^6^Department of Chemistry, Faculty of Science and Engineering, Swansea University, Swansea, UK

Email: [xiaohe.t@wchscu.cn](mailto:xiaohe.t@wchscu.cn), [haslina_ahmad@upm.edu.my](mailto:haslina_ahmad@upm.edu.my) and [m.r.gill@swansea.ac.uk](mailto:m.r.gill@swansea.ac.uk)

^‡^These authors contributed equally to the work.

**Supplementary information**

**Table of contents**

[Supplementary tables 2](#_Toc201848303)

[Supplementary figures 7](#_Toc201848304)

[Supplementary references 16](#_Toc201848305)

# Supplementary tables

**Supplementary Table S1. Details of the FDA-approved drugs set (166 compounds) obtained from the NCI.** NSC = Cancer Chemotherapy National Service Center number, USAN = United States Adopted Names and MW = molecular weight.

| PLATE | WELL ID | NSC | CAS | DRUG NAME (USAN) | MW (g/mol) |
| --- | --- | --- | --- | --- | --- |
| 4893 | A02 | 740 | 59-05-2 | Methotrexate | 454.44 |
| 4893 | B02 | 750 | 55-98-1 | Busulfan | 246.30 |
| 4893 | C02 | 752 | 154-42-7 | Thioguanine | 167.19 |
| 4893 | D02 | 755 | 50-44-2 | Mercaptopurine | 152.18 |
| 4893 | E02 | 762 | 55-86-7 | Mechlorethamine hydrochloride | 192.52 |
| 4893 | F02 | 1390 | 315-30-0 | Allopurinol | 136.11 |
| 4893 | G02 | 3053 | 50-76-0 | Dactinomycin | 1255.43 |
| 4893 | H02 | 3088 | 305-03-3 | Chlorambucil | 304.22 |
| 4893 | A03 | 6396 | 52-24-4 | Thiotepa | 189.22 |
| 4893 | B03 | 8806 | 3223-07-2 | Melphalan hydrochloride | 341.66 |
| 4893 | C03 | 9706 | 51-18-3 | Triethylenemelamine | 204.23 |
| 4893 | D03 | 13875 | 645-05-6 | Altretamine | 210.28 |
| 4893 | E03 | 18509 | 5451-09-2 | Aminolevulinic acid hydrochloride | 167.59 |
| 4893 | F03 | 19893 | 51-21-8 | Fluorouracil | 130.08 |
| 4893 | G03 | 24559 | 18378-89-7 | Plicamycin | 1085.16 |
| 4893 | H03 | 25154 | 54-91-1 | Pipobroman | 356.06 |
| 4893 | A04 | 26271 | 6055-19-2 | Cyclophosphamide | 261.09 |
| 4893 | B04 | 26980 | 50-07-7 | Mitomycin | 334.33 |
| 4893 | C04 | 27640 | 50-91-9 | Floxuridine | 246.19 |
| 4893 | D04 | 32065 | 127-07-1 | Hydroxyurea | 76.05 |
| 4893 | E04 | 34462 | 66-75-1 | Uracil mustard | 252.10 |
| 4893 | F04 | 38721 | 53-19-0 | Mitotane | 320.04 |
| 4893 | G04 | 45388 | 4342-03-4 | Dacarbazine | 182.18 |
| 4893 | H04 | 45923 | 298-81-7 | Methoxsalen | 216.19 |
| 4893 | A05 | 49842 | 143-67-9 | Vinblastine sulfate | 909.06 |
| 4893 | B05 | 63878 | 69-74-9 | Cytarabine hydrochloride | 279.70 |
| 4893 | C05 | 66847 | 50-35-1 | Thalidomide | 258.23 |
| 4893 | D05 | 67574 | 2068-78-2 | Vincristine sulfate | 923.04 |
| 4893 | E05 | 71423 | 595-33-5 | Megestrol acetate | 384.51 |
| 4893 | F05 | 75520 | 70-00-8 | Trifluridine | 296.20 |
| 4893 | G05 | 77213 | 366-70-1 | Procarbazine hydrochloride | 257.76 |
| 4893 | H05 | 79037 | 13010-47-4 | Lomustine | 233.70 |
| 4893 | A06 | 82151 | 23541-50-6 | Daunorubicin hydrochloride | 563.98 |
| 4893 | B06 | 85998 | 18883-66-4 | Streptozocin | 265.22 |
| 4893 | C06 | 92859 | 1327-53-3 | Arsenic trioxide | 197.84 |
| 4893 | D06 | 102816 | 320-67-2 | Azacitidine | 244.21 |
| 4893 | E06 | 105014 | 4291-63-8 | Cladribine | 285.69 |
| 4893 | F06 | 109724 | 3778-73-2 | Ifosfamide | 261.09 |
| 4893 | G06 | 119875 | 15663-27-1 | Cisplatin | 300.06 |
| 4893 | H06 | 122758 | 302-79-4 | Tretinoin | 300.44 |
| 4893 | A07 | 122819 | 29767-20-2 | Teniposide | 656.66 |
| 4893 | B07 | 123127 | 25316-40-9 | Doxorubicin hydrochloride | 579.99 |
| 4893 | C07 | 125066 | 9041-93-4 | Bleomycin sulfate | 1512.61 |
| 4893 | D07 | 125973 | 33069-62-4 | Paclitaxel | 853.92 |
| 4893 | E07 | 127716 | 2353-33-5 | Decitabine | 228.21 |
| 4893 | F07 | 138783 | 3543-75-7 | Bendamustine hydrochloride | 394.73 |
| 4893 | G07 | 141540 | 33419-42-0 | Etoposide | 588.56 |
| 4893 | H07 | 169780 | 24584-09-6 | Dexrazoxane | 268.27 |
| 4893 | A08 | 180973 | 54965-24-1 | Tamoxifen citrate | 563.65 |
| 4893 | B08 | 218321 | 53910-25-1 | Pentostatin | 268.27 |
| 4893 | C08 | 226080 | 53123-88-9 | Sirolimus | 914.18 |
| 4893 | D08 | 241240 | 41575-94-4 | Carboplatin | 371.25 |
| 4893 | E08 | 246131 | 56124-62-0 | Valrubicin | 723.64 |
| 4893 | F08 | 256439 | 57852-57-0 | Idarubicin hydrochloride | 533.96 |
| 4893 | G08 | 256942 | 56390-09-1 | Epirubicin hydrochloride | 579.99 |
| 4893 | H08 | 266046 | 61825-94-3 | Oxaliplatin | 397.29 |
| 4893 | A09 | 279836 | 65271-80-9 | Mitoxantrone | 444.49 |
| 4893 | B09 | 296961 | 20537-88-6 | Amifostine | 214.22 |
| 4893 | C09 | 312887 | 75607-67-9 | Fludarabine phosphate | 365.21 |
| 4893 | D09 | 362856 | 85622-93-1 | Temozolomide | 194.15 |
| 4893 | E09 | 369100 | 99011-02-6 | Imiquimod | 240.31 |
| 4893 | F09 | 409962 | 154-93-8 | Carmustine | 214.05 |
| 4893 | G09 | 606869 | 123318-82-1 | Clofarabine | 303.68 |
| 4893 | H09 | 608210 | 125317-39-7 | Vinorelbine tartrate | 1079.00 |
| 4893 | A10 | 609699 | 119413-54-6 | Topotecan hydrochloride | 457.91 |
| 4893 | B10 | 613327 | 122111-03-9 | Gemcitabine hydrochloride | 299.65 |
| 4893 | C10 | 616348 | 100286-90-6 | Irinotecan hydrochloride | 623.15 |
| 4893 | D10 | 628503 | 114977-28-5 | Docetaxel | 807.89 |
| 4893 | E10 | 683864 | 162635-04-3 | Temsirolimus | 1030.29 |
| 4893 | F10 | 701852 | 149647-78-9 | Vorinostat | 264.32 |
| 4893 | G10 | 702294 | 52205-73-9 | Estramustine phosphate sodium | 564.35 |
| 4893 | H10 | 712807 | 154361-50-9 | Capecitabine | 359.35 |
| 4893 | A11 | 713563 | 107868-30-4 | Exemestane | 296.40 |
| 4893 | B11 | 715055 | 184475-35-2 | Gefitinib | 446.90 |
| 4893 | C11 | 718781 | 183319-69-9 | Erlotinib hydrochloride | 429.90 |
| 4893 | D11 | 719276 | 129453-61-8 | Fulvestrant | 606.75 |
| 4893 | E11 | 719344 | 120511-73-1 | Anastrozole | 293.37 |
| 4893 | F11 | 719345 | 112809-51-5 | Letrozole | 285.30 |
| 4893 | G11 | 719627 | 169590-42-5 | Celecoxib | 381.37 |
| 4893 | H11 | 721517 | 118072-93-8 | Zoledronic acid | 272.09 |
| 4894 | A02 | 732517 | 863127-77-9 | Dasatinib | 488.01 |
| 4894 | B02 | 733504 | 159351-69-6 | Everolimus | 958.24 |
| 4894 | C02 | 737754 | 635702-64-6 | Pazopanib hydrochloride | 473.98 |
| 4894 | D02 | 741078 | 606143-52-6 | Selumetinib | 457.68 |
| 4894 | E02 | 743414 | 152459-95-5 | Imatinib | 493.61 |
| 4894 | F02 | 745750 | 231277-92-2 | Lapatinib | 581.06 |
| 4894 | G02 | 747599 | 641571-10-0 | Nilotinib | 529.51 |
| 4894 | H02 | 747971 | 284461-73-0 | Sorafenib | 464.82 |
| 4894 | A03 | 747972 | 191732-72-6 | Lenalidomide | 259.26 |
| 4894 | B03 | 747973 | 219989-84-1 | Ixabepilone | 506.70 |
| 4894 | C03 | 747974 | 84449-90-1 | Raloxifene | 473.59 |
| 4894 | D03 | 749226 | 154229-19-3 | Abiraterone | 349.51 |
| 4894 | E03 | 750690 | 557795-19-4 | Sunitinib | 398.47 |
| 4894 | F03 | 750691 | 439081-18-2 | Afatinib | 485.94 |
| 4894 | G03 | 753686 | 763113-22-0 | Olaparib | 434.46 |
| 4894 | H03 | 754143 | 128517-07-7 | Romidepsin | 540.69 |
| 4894 | A04 | 754230 | 146464-95-1 | Pralatrexate | 477.48 |
| 4894 | B04 | 754355 | 1038915-60-4 | Niraparib hydrochloride | 356.85 |
| 4894 | C04 | 755384 | 357166-30-4 | Pemetrexed, Disodium salt, Heptahydrate | 471.38 |
| 4894 | D04 | 755605 | 915087-33-1 | Enzalutamide | 464.42 |
| 4894 | E04 | 755980 | 417716-92-8 | Lenvatinib | 426.86 |
| 4894 | F04 | 755985 | 121032-29-9 | Nelarabine | 297.27 |
| 4894 | G04 | 755986 | 879085-55-9 | Vismodegib | 421.30 |
| 4894 | H04 | 756644 | 459868-92-9 | Rucaparib phosphate | 421.36 |
| 4894 | A05 | 756645 | 877399-52-5 | Crizotinib | 450.34 |
| 4894 | B05 | 756655 | 179324-69-7 | Bortezomib | 384.24 |
| 4894 | C05 | 757439 | 698387-09-6 | Neratinib | 557.05 |
| 4894 | D05 | 757441 | 319460-85-0 | Axitinib | 386.47 |
| 4894 | E05 | 758246 | 871700-17-3 | Trametinib | 615.40 |
| 4894 | F05 | 758247 | 571190-30-2 | Palbociclib | 447.54 |
| 4894 | G05 | 758252 | 868540-17-4 | Carfilzomib | 719.92 |
| 4894 | H05 | 758253 | 26833-87-4 | Omacetaxine mepesuccinate | 545.63 |
| 4894 | A06 | 758254 | 1201902-80-8 | Ixazomib citrate | 517.13 |
| 4894 | B06 | 758487 | 943319-70-8 | Ponatinib | 532.55 |
| 4894 | C06 | 758774 | 414864-00-9 | Belinostat | 318.35 |
| 4894 | D06 | 759224 | 870281-82-6 | Idelalisib | 415.42 |
| 4894 | E06 | 760766 | 443913-73-3 | Vandetanib | 475.36 |
| 4894 | F06 | 761068 | 849217-68-1 | Cabozantinib | 501.51 |
| 4894 | G06 | 761190 | 404950-80-7 | Panobinostat | 349.43 |
| 4894 | H06 | 761385 | 956697-53-3 | Erismodegib | 485.49 |
| 4894 | A07 | 761388 | 110078-46-1 | Plerixafor | 502.79 |
| 4894 | B07 | 761431 | 1029872-54-5 | Vemurafenib | 489.91 |
| 4894 | C07 | 761432 | 183133-96-2 | Cabazitaxel | 835.94 |
| 4894 | D07 | 761910 | 936563-96-1 | Ibrutinib | 440.50 |
| 4894 | E07 | 763371 | 941678-49-5 | Ruxolitinib | 306.36 |
| 4894 | F07 | 763932 | 755037-03-7 | Regorafenib | 482.80 |
| 4894 | G07 | 764040 | 1256580-46-7 | Alectinib | 482.62 |
| 4894 | H07 | 764042 | 606143-89-9 | Binimetinib | 441.22 |
| 4894 | A08 | 764134 | 1195768-06-9 | Dabrafenib mesylate | 615.65 |
| 4894 | B08 | 764581 | 937263-43-9 | ARRY-380 | 480.52 |
| 4894 | C08 | 765694 | 380843-75-4 | Bosutinib | 530.45 |
| 4894 | D08 | 765888 | 1110813-31-4 | Dacomitinib | 469.94 |
| 4894 | E08 | 765974 | 1217486-61-7 | Alpelisib | 441.47 |
| 4894 | F08 | 766270 | 1257044-40-8 | Venetoclax | 868.45 |
| 4894 | G08 | 767125 | 1207456-01-6 | Talazoparib | 380.35 |
| 4894 | H08 | 767600 | 936091-26-8 | Fedratinib | 524.67 |
| 4894 | A09 | 768068 | 934660-93-2 | Cobimetinib | 531.32 |
| 4894 | B09 | 768073 | 1231929-97-7 | Abemaciclib | 506.59 |
| 4894 | C09 | 771649 | 956104-40-8 | Apalutamide | 477.42 |
| 4894 | D09 | 772469 | 1201438-56-3 | Duvelisib | 416.87 |
| 4894 | E09 | 774769 | 1108743-60-7 | Entrectinib | 560.63 |
| 4894 | F09 | 775351 | 19171-19-8 | Pomalidomide | 273.25 |
| 4894 | G09 | 775772 | 1095173-27-5 | Glasdegib | 374.43 |
| 4894 | H09 | 776422 | 1032900-25-6 | Ceritinib | 558.14 |
| 4894 | A10 | 777109 | 1403254-99-8 | Tazemetostat | 572.73 |
| 4894 | B10 | 777878 | 1029712-80-8 | Capmatinib | 412.41 |
| 4894 | C10 | 778304 | 1269440-17-6 | Encorafenib | 540.01 |
| 4894 | D10 | 778909 | 1211441-98-3 | Ribociclib | 434.54 |
| 4894 | E10 | 779217 | 1421373-65-0 | Osimertinib | 499.61 |
| 4894 | F10 | 780108 | 1454846-35-5 | Lorlatinib | 406.41 |
| 4894 | G10 | 780203 | 1393477-72-9 | Selinexor | 443.30 |
| 4894 | H10 | 781556 | 1346242-81-6 | Erdafitinib | 446.54 |
| 4894 | A11 | 785570 | 1223403-58-4 | Larotrectinib | 428.43 |
| 4894 | B11 | 787457 | 1197953-54-0 | Brigatinib | 584.10 |
| 4894 | C11 | 787846 | 1254053-43-4 | Gilteritinib | 552.71 |
| 4894 | D11 | 788120 | 1446502-11-9 | Enasidenib | 473.35 |
| 4894 | E11 | 788948 | 4105-38-8 | Uridine triacetate | 370.32 |
| 4894 | F11 | 789102 | 1448347-49-6 | Ivosidenib | 582.96 |
| 4894 | G11 | 789300 | 1029044-16-3 | Pexidartinib | 417.81 |
| 4894 | H11 | 791164 | 1420477-60-6 | Acalabrutinib | 465.41 |
| 4895 | A02 | 801082 | 1703793-34-3 | Avapritinib | 498.55 |
| 4895 | B02 | 816437 | N/A | Copanlisib tris-HCl | 589.90 |
| 4895 | C02 | 816556 | 1513857-77-6 | Pemigatinib | 487.49 |
| 4895 | D02 | 818434 | 2152628-33-4 | Selpercatinib | 525.60 |
| 4895 | E02 | 823807 | 1691249-45-2 | Zanubrutinib | 471.55 |
| 4895 | F02 | 825331 | 1297538-32-9 | Darolutamide | 398.84 |

**Supplementary Table S2. Combination indices (CIs) for the combinations of the library compound with Olaparib in MDA-MB-231 cells for 24 h treatment.** Sub-cytotoxic concentration of Olaparib (10 μM) was used in the combination treatment.


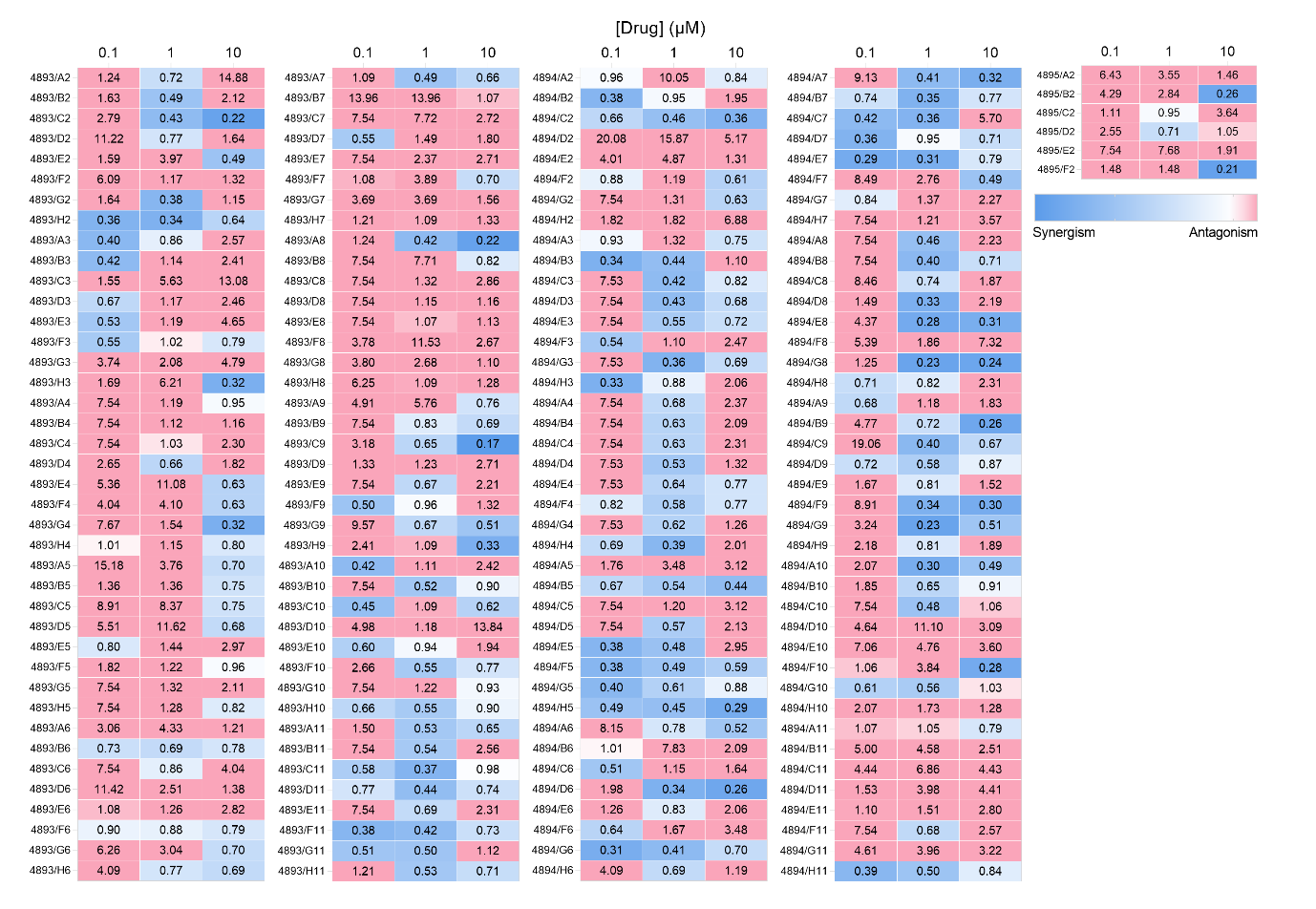


**Supplementary Table S3. IC_50_ values of each compound alone or in combination with Olaparib (10 μM) in MDA-MB-231 cells, 24 h treatment, as determined from MTT data**. Data expressed as mean ± SD of two independent experiments. Fold shift = IC_50_ (- OLAP)/IC_50_ (+ OLAP). ND = not determined.

| Compound | | 24 h IC_50_ (µM) | | Fold Change |
| --- | --- | --- | --- | --- |
|  |  | - 10 µM OLAP | + 10 µM OLAP |  |
| 4893/49_H2 | Chlorambucil | >100 | 60.0 ± 12.4 | > 1.6 |
| 4893/49_A8 | Tamoxifen Citrate | >100 | 15.4 ± 0.9 | > 6.4 |
| 4893/49_C9 | Fludarabine Phosphate | >100 | 0.5 ± 0.1 | > 200 |
| 4893/49_A11 | Exemestane | >100 | 16.8 ± 12.5 | > 5.9 |
| 4893/49_H11 | Zoledronic acid | >100 | 51.9 ± 0.9 | > 1.9 |
| 4894/49_D3 | Abiraterone | >100 | 21.8 ± 10.4 | > 4.5 |
| 4894/49_H5 | Omacetaxine mepesuccinate | 1.6 ± 1.0 | 0.2 ± 0.1 | 8 |
| 4894/49_G6 | Panobinostat | 4.1 ± 2.8 | 0.1 ± 0.0 | 41 |
| 4894/49_A7 | Plerixafor | >100 | 66.6 ± 11.6 | > 1.5 |
| 4894/49_H11 | Acalabrutinib | >100 | >100 | ND |

**Supplementary Table S4. Detail of the ten hit compounds including their drug classes, human safety profiles of these drugs and their PARPi combinations status.** NA = Not Available.

| Compound | Drug class | Human safety profiles | PARPi combination |
| --- | --- | --- | --- |
| Chlorambucil | DNA alkylating agent | Severe toxicity | NA |
| Tamoxifen citrate | a selective oestrogen receptor modulator | NA | [1] |
| Fludarabine phosphate | a purine analogue antimetabolite | Severe toxicity | NA |
| Exemestane | aromatase inhibitor | NA | NA |
| Zoledronic acid | bisphosphonate | NA | NA |
| Abiraterone | antiandrogen | NA | [2] |
| Omacetaxine mepesuccinate | cephalotaxine | NA | NA |
| Panobinostat | non-selective histone deacetylase inhibitor | Severe toxicity | NA |
| Plerixafor | a selective chemokine receptor (CXCR4) antagonist | NA | NA |
| Acalabrutinib | a Bruton tyrosine kinase inhibitor | NA | NA |

**Supplementary Table S5.** **IC_50_ values of Exemestane alone or in combination with Olaparib (10 μM) in MDA-MB-436 and MCF7 cells, 24 h treatment, as determined from MTT data**. Data expressed as mean ± SD of two independent experiments. Fold shift = IC_50_ (- OLAP)/IC_50_ (+ OLAP). Data for MDA-MB-231 cells are included from Supplementary Table S4 for comparison.

|  | **24 h IC_50_ of EXE (µM)** | | **Fold Change** |
| --- | --- | --- | --- |
|  | **- OLAP** | **+ OLAP** |  |
| MDA-MB-231 | >100 | 16.8 ± 12.5 | > 5.9 |
| MDA-MB-436 | > 100 | 93.23 $\pm$ 5.24 | > 1.07 |
| HCC1937 | > 100 | 91.05 0.79 | > 1.09 |
| MCF7 | 88.09 $\pm$ 2.13 | 57.20 $\pm$ 9.07 | 1.54 |
| MCF10A | > 100 | > 100 | ND |

# Supplementary figures


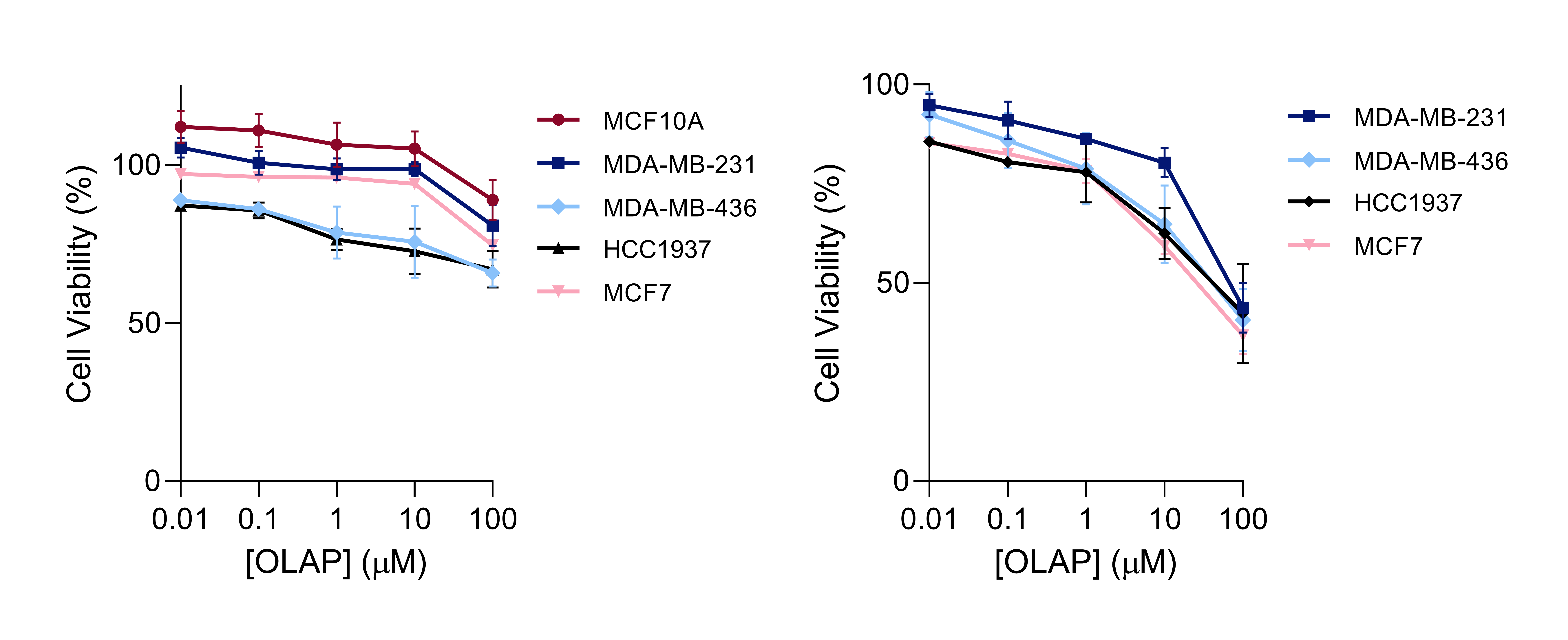


**Supplementary Fig. S1 Cell viability of MCF10A normal breast, MDA-MB-231 TNBC, MDA-MB-436 TNBC, HCC1937 TNBC and MCF7 breast cancer cells following treatment with concentration gradient of Olaparib for 24 h (left) or 72 h (right), as determined by MTT assay.** Data were expressed as mean ± SD of three independent experiments.


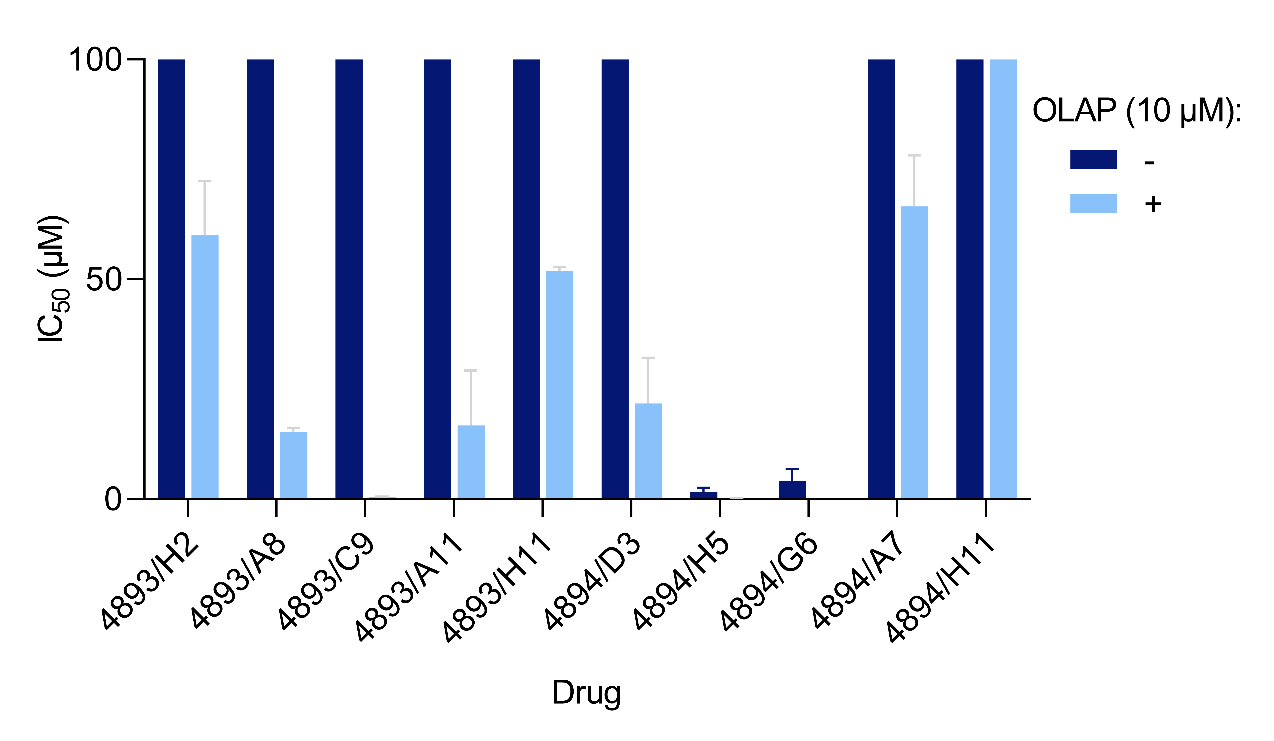


**Supplementary Fig. S2 IC_50_ values of each compound alone or in combination with Olaparib (10 μM) in MDA-MB-231 cells, 24 h treatment, as determined from MTT data**. Data expressed as mean ± SD of two independent experiments.


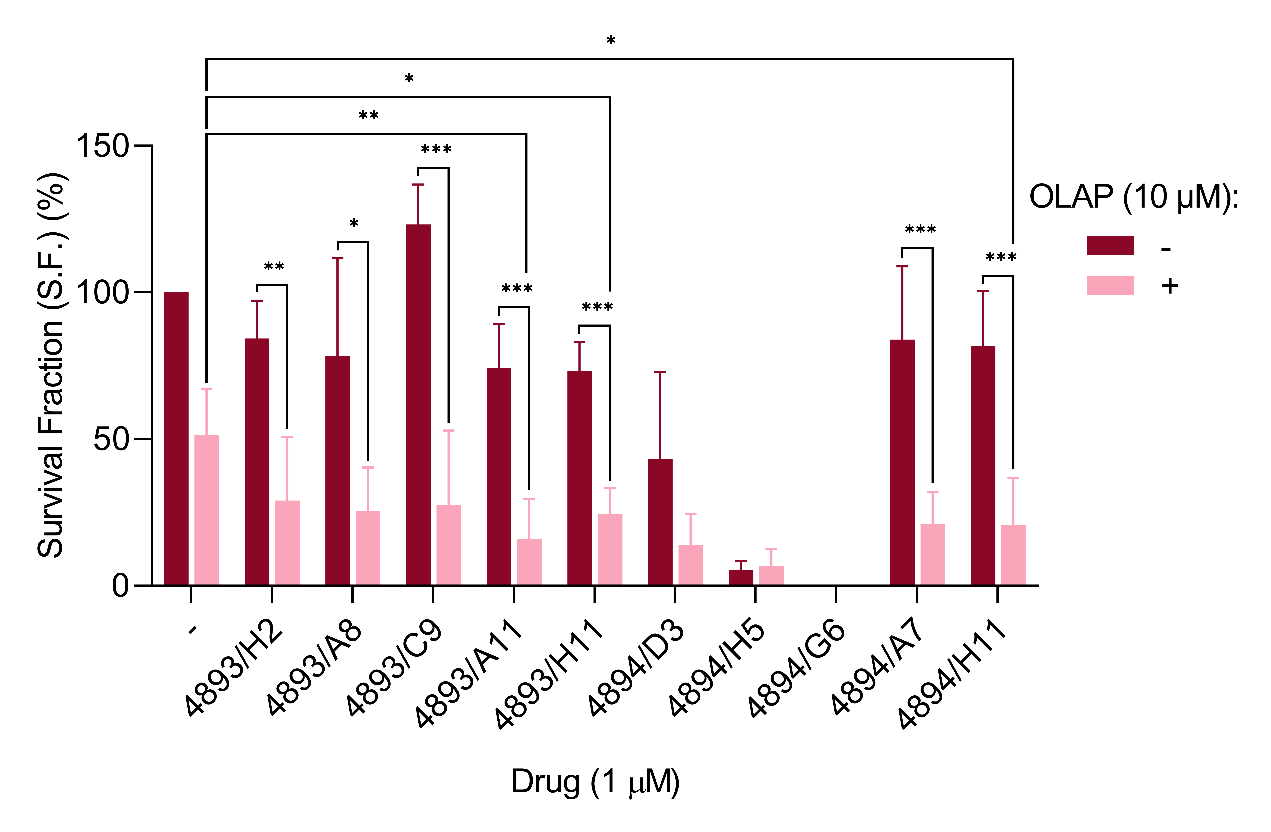


**Supplementary Fig. S3 Clonogenic survival assay of MDA-MB-231 cells treated with the stated single-agent (1 µM), Olaparib (10 µM) or both for 24 h.** Data were expressed as mean $\pm$ SD of four independent experiments (n = 4). **P* < 0.05, ***P* < 0.01 and ****P* < 0.001 compared to single agent-treated groups by ANOVA.


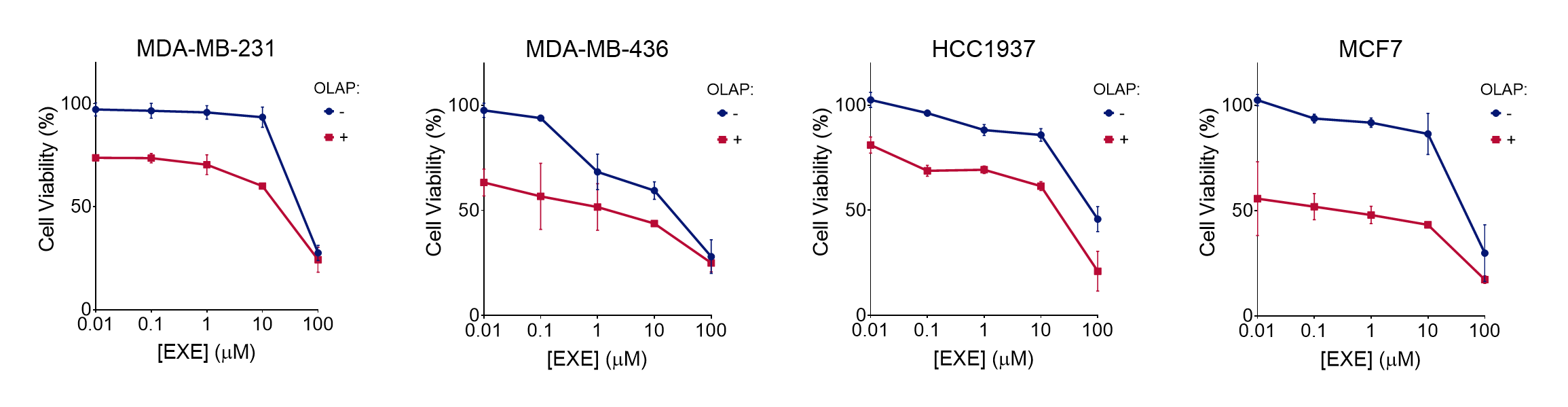


**Supplementary Fig. S4 Cell viability of MDA-MB-231 TNBC, MDA-MB-436 TNBC, HCC1937 TNBC and MCF7 breast cancer cells following treatment with concentration gradient of Olaparib for 72 h, as determined by MTT assay.** Data were expressed as mean ± SD of three independent experiments.


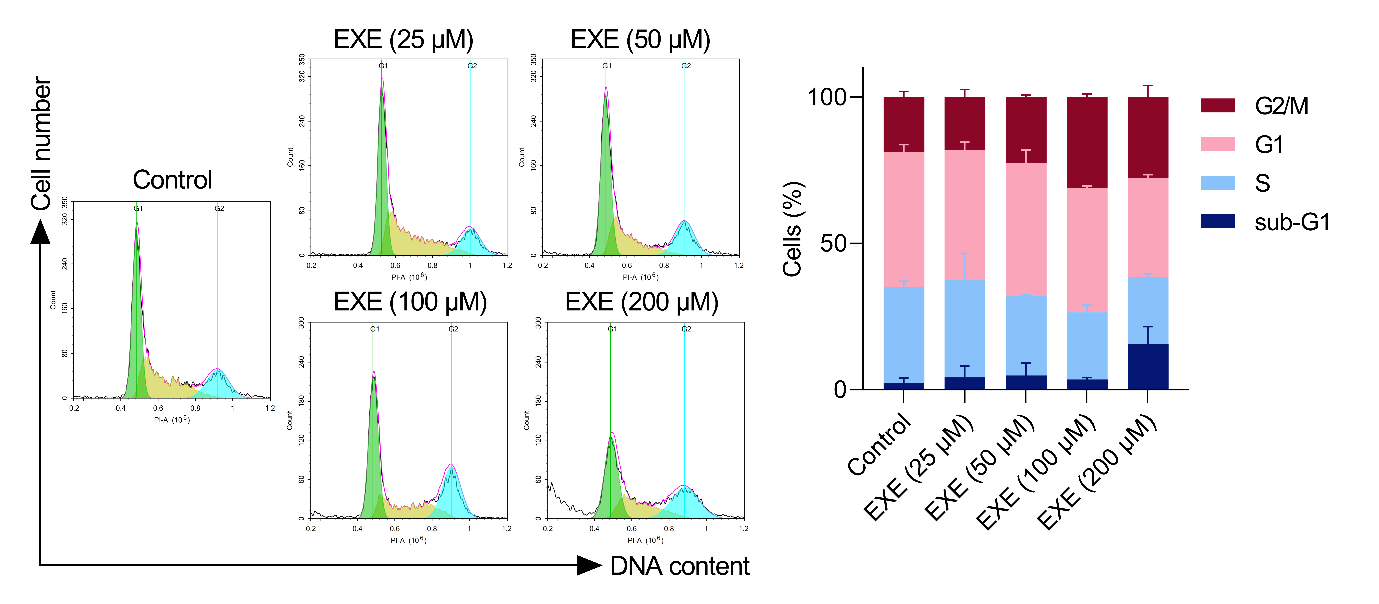
 **Supplementary Fig. S5 Cell cycle distribution following 24 h treatment with Exemestane (25, 50, 100 and 200 µM, as determined by PI staining and flow cytometry.** Left, representative histograms, right, quantification of cell cycle phase.


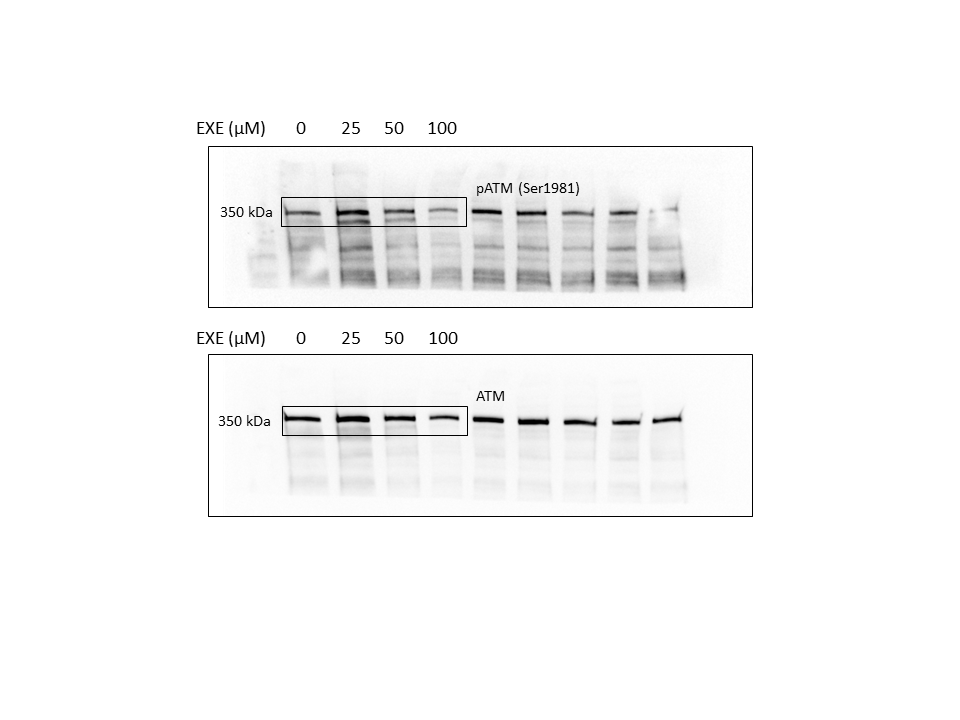

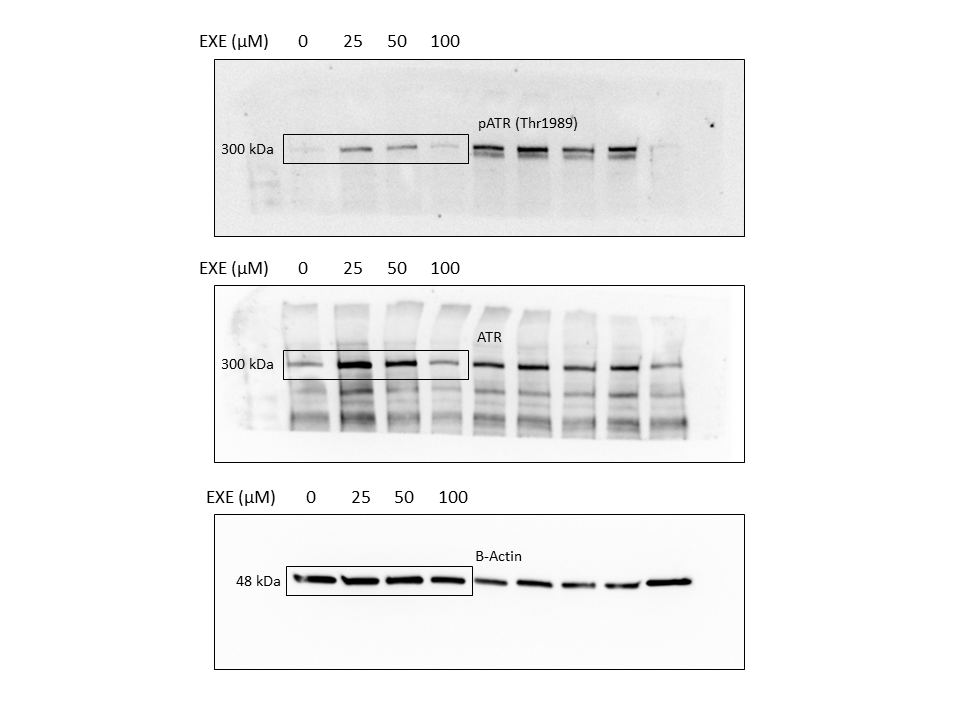

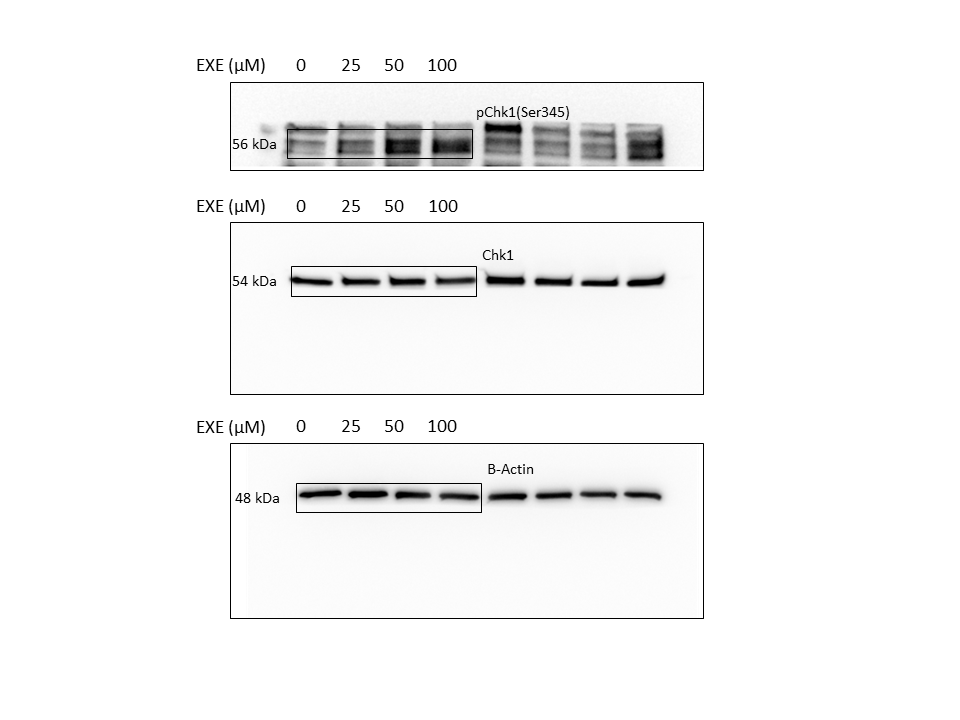


**Supplementary Fig. S6 Uncropped blot images from Fig. 3 with molecular weight (kDa) of the protein of interest on the left.**


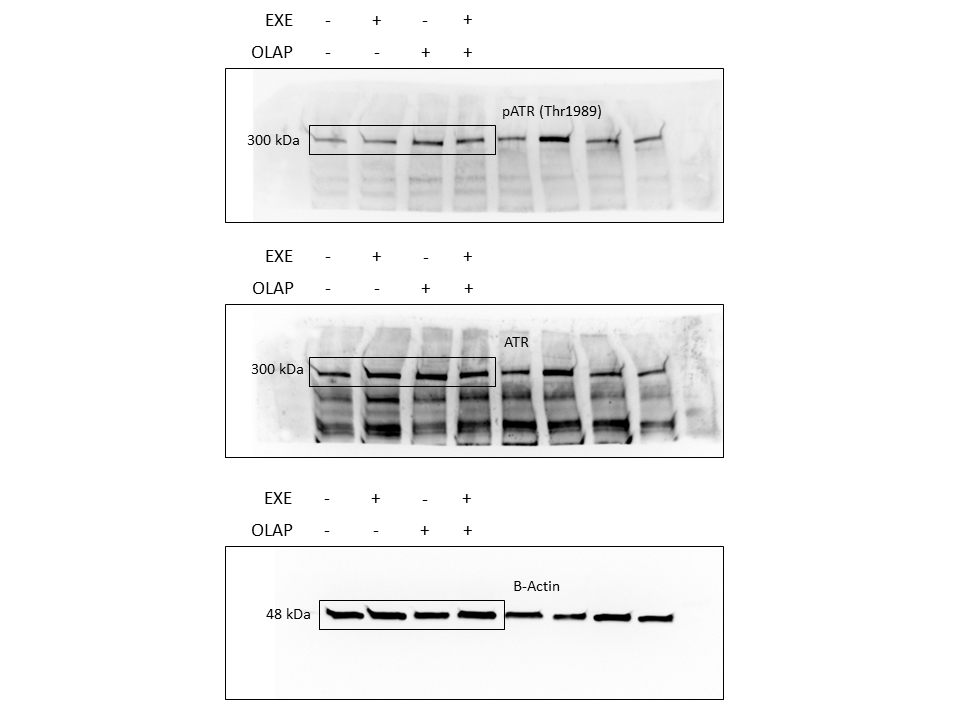

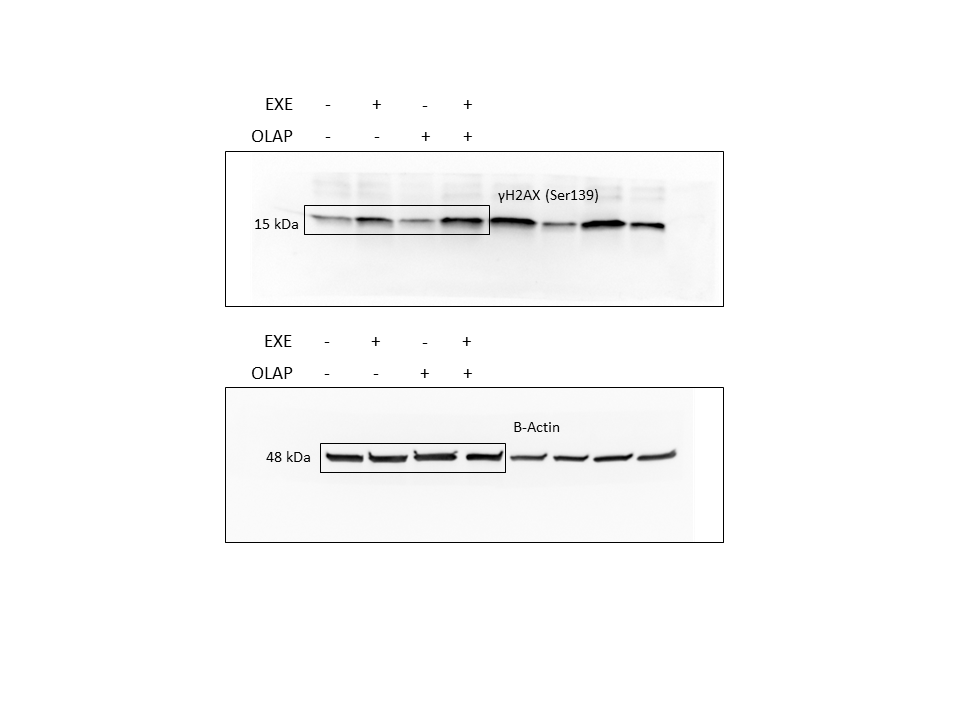


**Supplementary Fig. S7 Uncropped blot images from Fig. 4 with molecular weight (kDa) of the protein of interest on the left.**


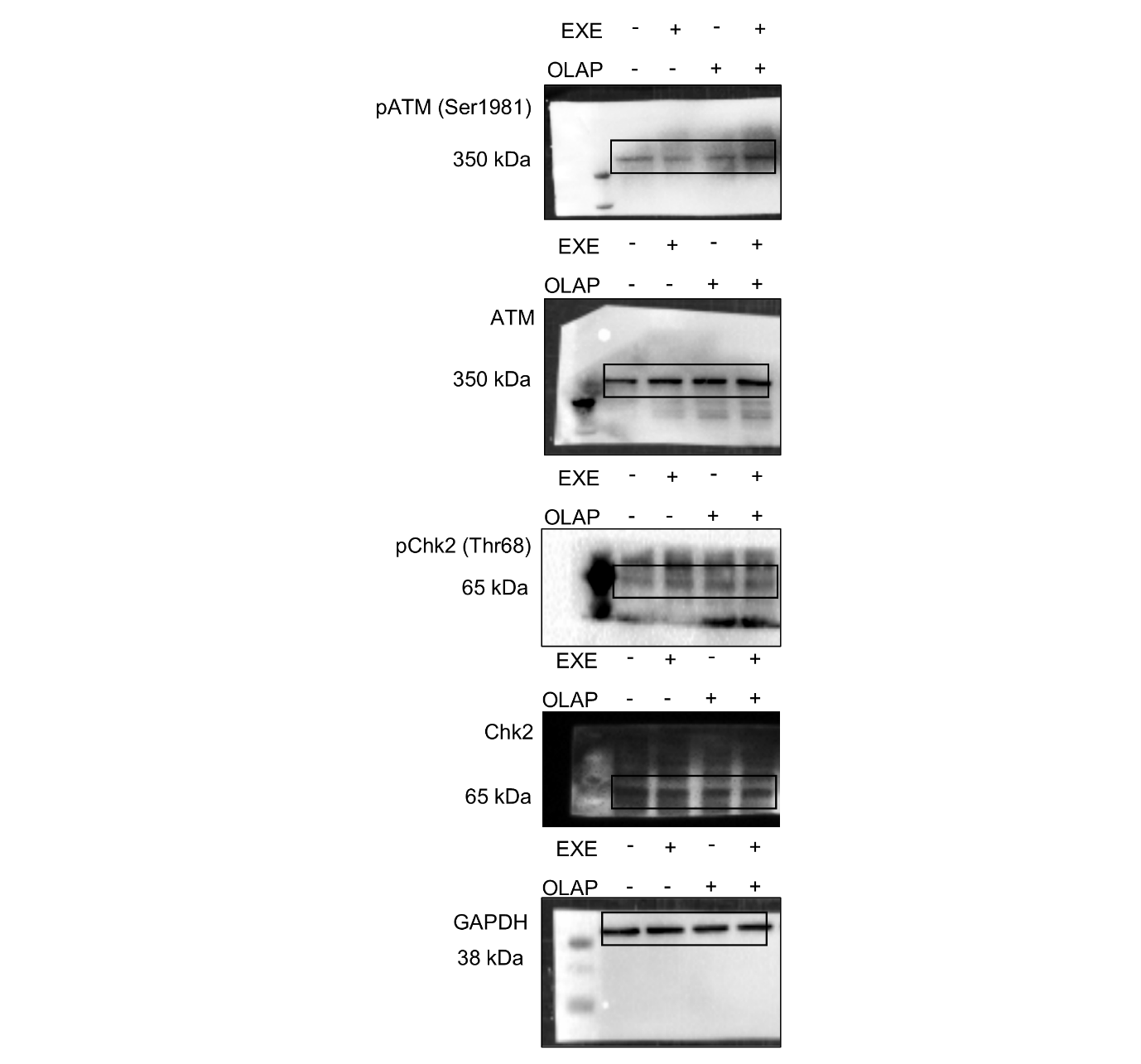


**Supplementary Fig. S8 Uncropped blot images from Fig. 4 with molecular weight (kDa) of the protein of interest on the left.**


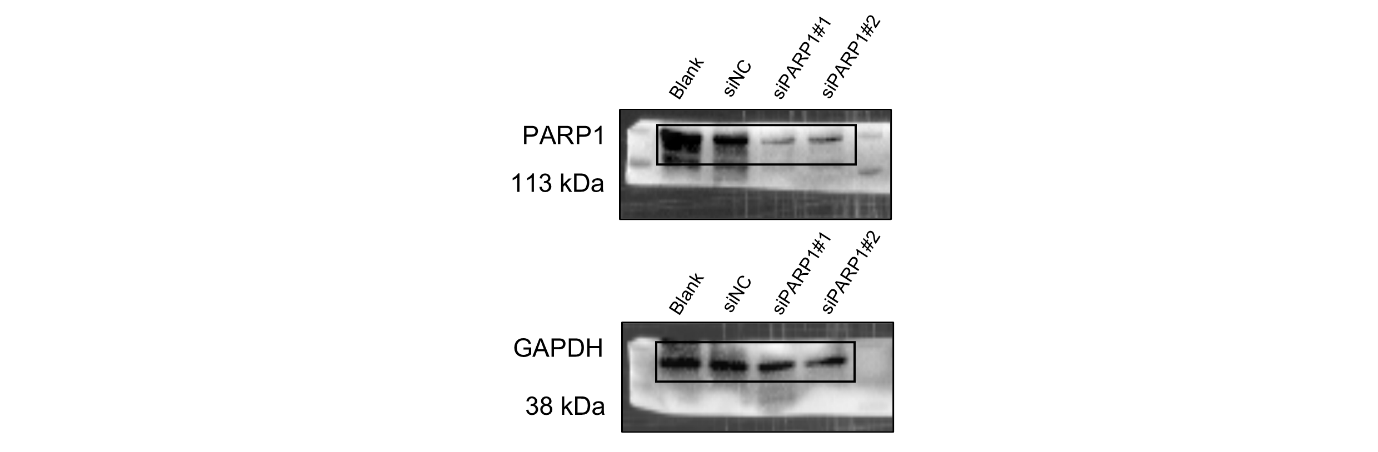


**Supplementary Fig. S9 Uncropped blot images from Fig. 5 with molecular weight (kDa) of the protein of interest on the left.**


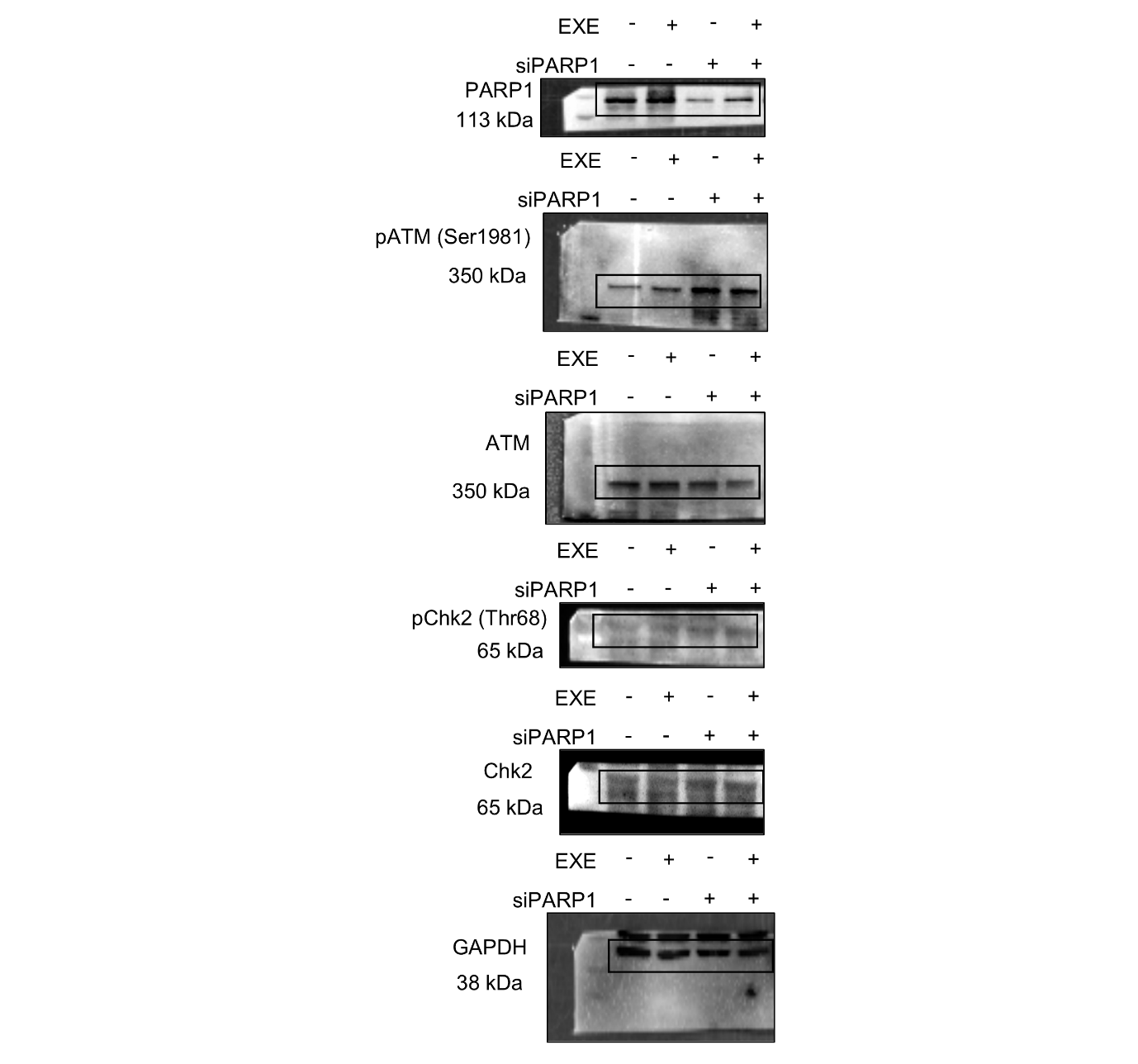


**Supplementary Fig. S10 Uncropped blot images from Fig. 5 with molecular weight (kDa) of the protein of interest on the left.**


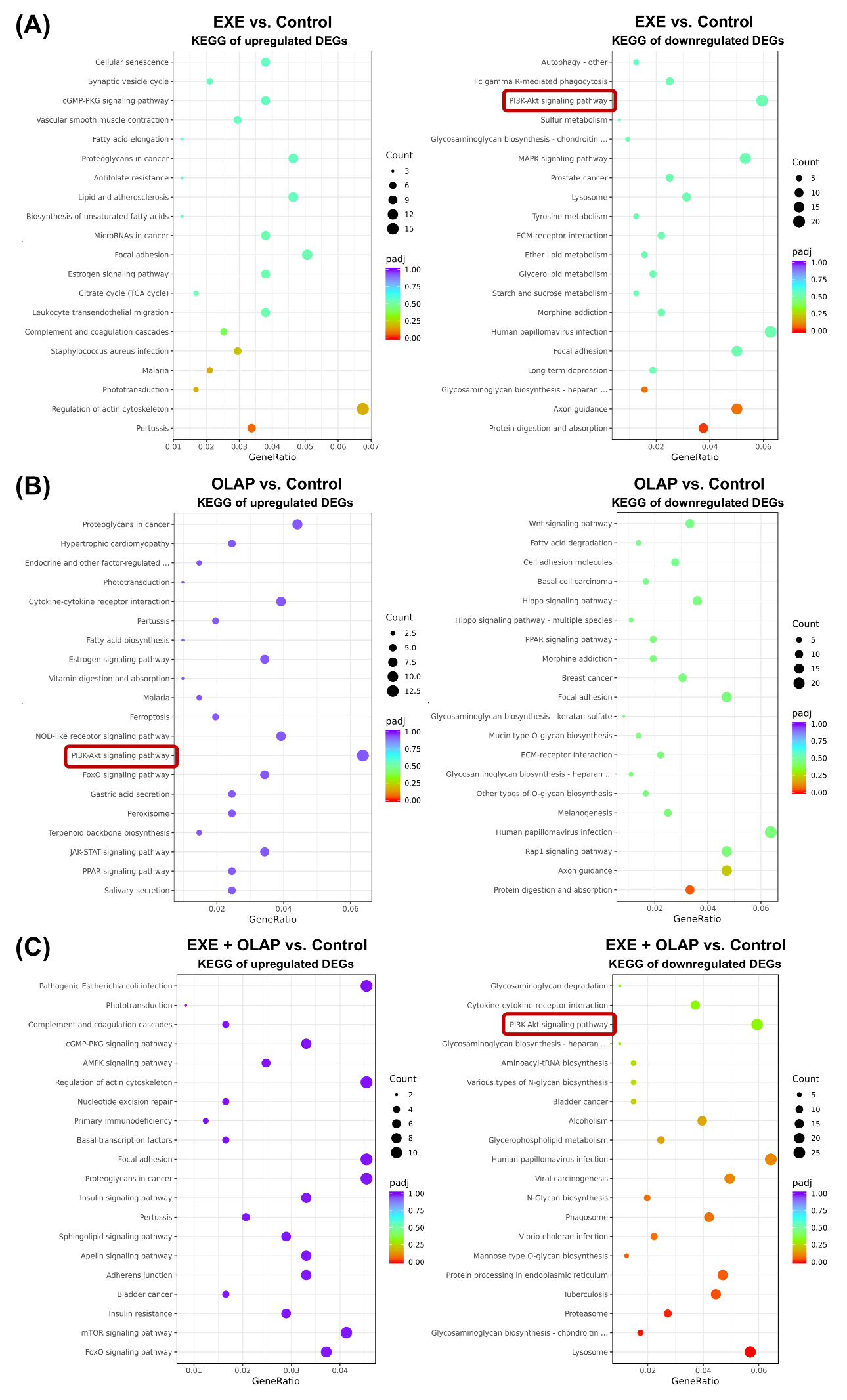


**Supplementary Fig. S11 KEGG pathway enrichment analysis of DEGs in transcriptomes of (A) EXE-, (B) OLAP-, and (C) EXE+OLAP-treated groups in comparison to control group.** The top 20 significantly enriched pathways are presented in order of enrichment score. Dot size represents gene count, and colour represents the *P* value. *P* < 0.05 is considered statistically significant.


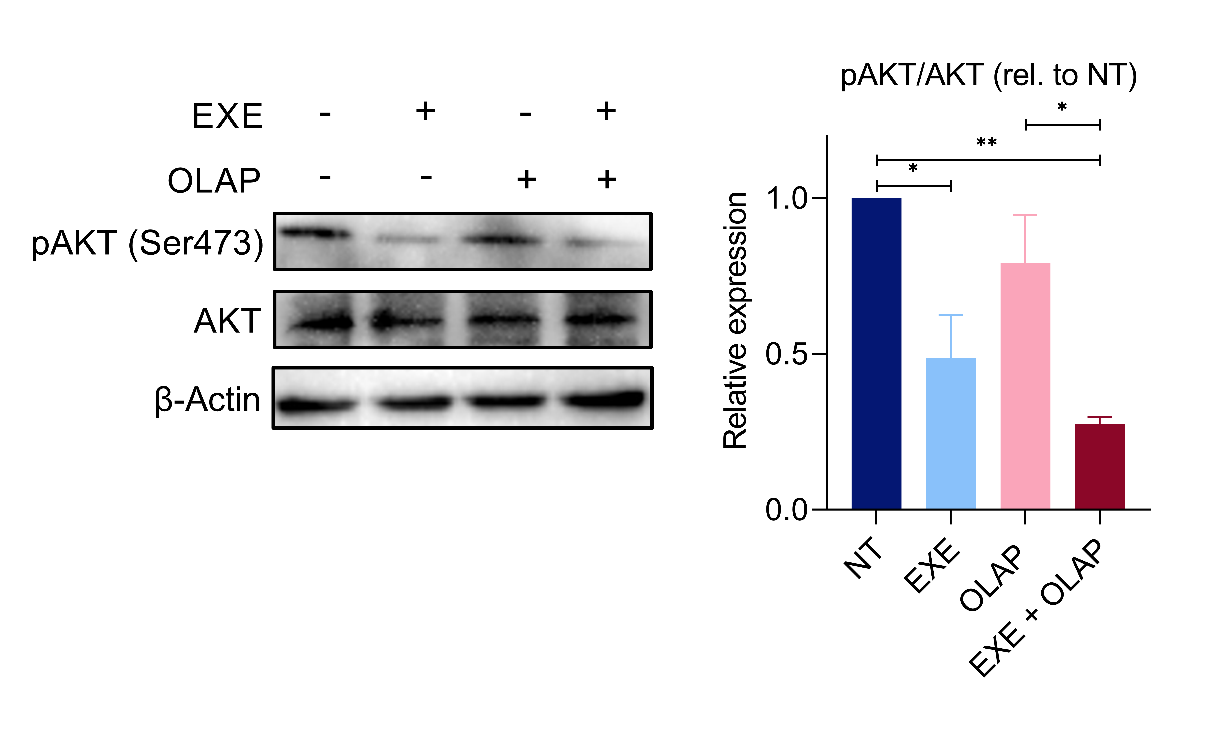


**Supplementary Fig. S12 Western blot analysis of AKT signalling pathway following treatment with Exemestane (25 µM) and Olaparib (10 µM) for 3 h.** Left, western blot images, right, quantification of protein bands from western blot images by densitometry. β-actin levels were monitored as a loading control. NT = untreated control. Uncropped blot images are shown in Supplementary Fig. S13. Data expressed as mean ± SD of two independent experiments. **P* < 0.05 and ***P* < 0.01 by ANOVA.


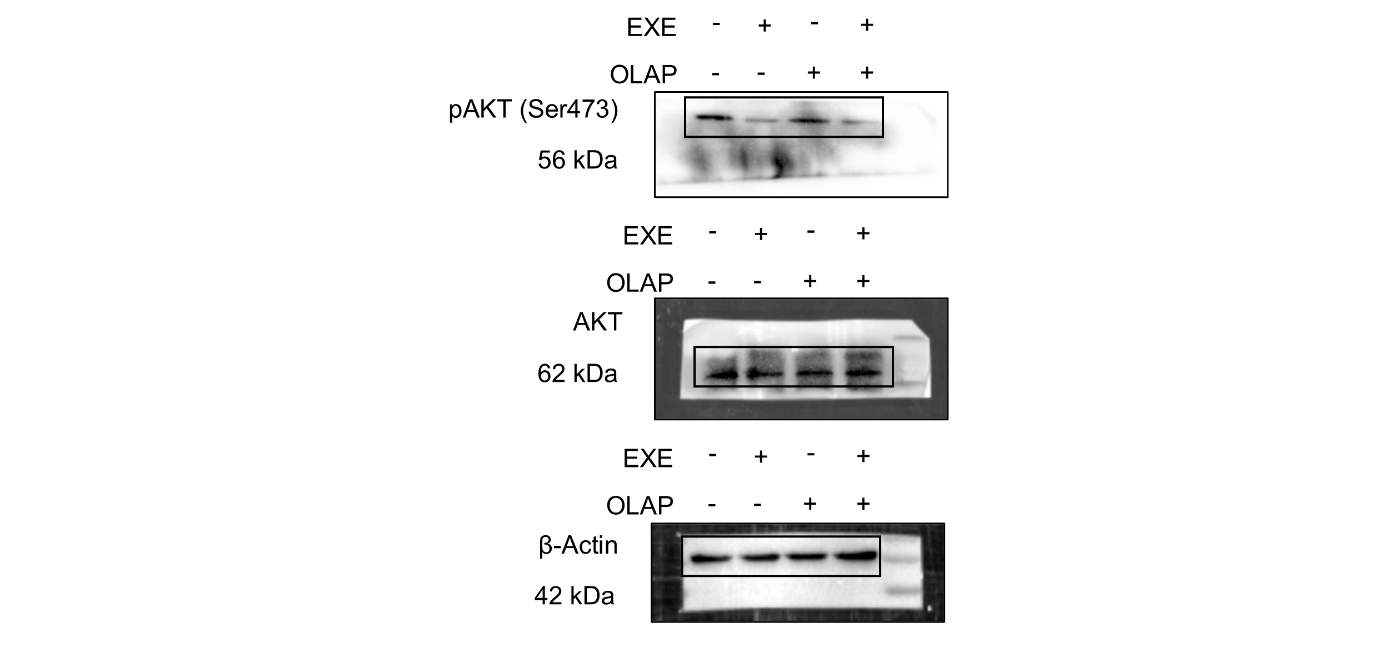


**Supplementary Fig. S13 Uncropped blot images from Supplementary Fig. S10 with molecular weight (kDa) of the protein of interest on the left.**


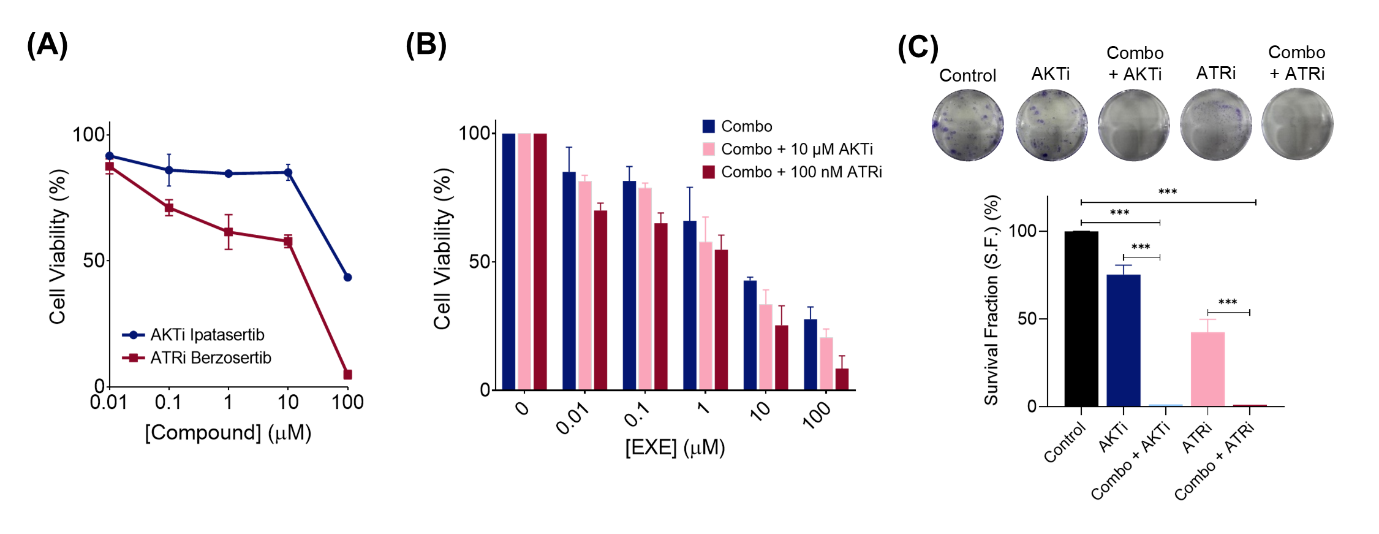


**Supplementary Fig. S14** **AKT and ATR inhibitors enhance the cytotoxic effect of Exemestane and Olaparib in MDA-MB-231 cells.** (A) Cell viability of MDA-MB-231 cells following 24 h treatment with increasing concentrations of the AKTi Ipatasertib and the ATRi Berzosertib, assessed by MTT assay. (B) Cell viability of MDA-MB-231 cells treated for 24 h with concentration gradients of Exemestane in combination with 10 µM Olaparib and either 10 µM AKTi or 100 nM ATRi, as measured by MTT assay. (B) Clonogenic survival assay of MDA-MB-231 cells treated for 24 h with Exemestane (25 µM), Olaparib (10 µM), AKTi (10 µM), and ATRi (100 nM) alone or in combination. Left panels show representative colony images; right panels show quantification of survival fractions. ****P* < 0.001 by ANOVA.

# Supplementary references

1. Plummer R, Verheul HM, De Vos F, Leunen K, Molife LR, Rolfo C, Grundtvig-Sørensen P, De Grève J, Rottey S, Jerusalem G, Italiano A, Spicer J, Dirix L, Goessl C, Birkett J, Spencer S, Learoyd M, Bailey C & Dean E (2018) Pharmacokinetic Effects and Safety of Olaparib Administered with Endocrine Therapy: A Phase I Study in Patients with Advanced Solid Tumours. *Adv Ther* **35**(11), 1945-1964.

2. Clarke NW, Armstrong AJ, Thiery-Vuillemin A, Oya M, Shore N, Loredo E, Procopio G, Menezes Jd, Girotto G, Arslan C, Mehra N, Parnis F, Brown E, Schlürmann F, Joung JY, Sugimoto M, Virizuela JA, Emmenegger U, Navratil J, Buchschacher GL, Poehlein C, Harrington EA, Desai C, Kang J & Saad F (2022) Abiraterone and Olaparib for Metastatic Castration-Resistant Prostate Cancer. *NEJM Evidence* **1**(9), EVIDoa2200043.
